# Designer cells programming quorum-sensing interference with microbes

**DOI:** 10.1038/s41467-018-04223-7

**Published:** 2018-05-08

**Authors:** Ferdinand Sedlmayer, Dennis Hell, Marius Müller, David Ausländer, Martin Fussenegger

**Affiliations:** 1 Department of Biosystems Science and Engineering, ETH Zürich, Mattenstrasse 26,, CH-4058 Basel, Switzerland; 2 Faculty of Science, University of Basel, Mattenstrasse 26, CH-4058 Basel, Switzerland

## Abstract

Quorum sensing is a promising target for next-generation anti-infectives designed to address evolving bacterial drug resistance. The autoinducer-2 (AI-2) is a key quorum-sensing signal molecule which regulates bacterial group behaviors and is recognized by many Gram-negative and Gram-positive bacteria. Here we report a synthetic mammalian cell-based microbial-control device that detects microbial chemotactic formyl peptides through a formyl peptide sensor (FPS) and responds by releasing AI-2. The microbial-control device was designed by rewiring an artificial receptor-based signaling cascade to a modular biosynthetic AI-2 production platform. Mammalian cells equipped with the microbial-control gene circuit detect formyl peptides secreted from various microbes with high sensitivity and respond with robust AI-2 production, resulting in control of quorum sensing-related behavior of pathogenic *Vibrio harveyi* and attenuation of biofilm formation by the human pathogen *Candida albicans*. The ability to manipulate mixed microbial populations through fine-tuning of AI-2 levels may provide opportunities for future anti-infective strategies.

## Abstract (web summary)

Bacterial populations communicate with AI-2 signaling molecules, helping to coordinate biofilm development and other group behaviors. Here the authors design a genetic circuit for mammalian cells that allows them to sense bacterial populations and interfere with quorum communication.

## Introduction

Synthetic biology uses engineering principles to introduce desirable functionalities into biological systems by rewiring standardized biological parts in a rational and predictable manner^1,2^. By implementing gene circuits that interfere with pathological responses in metabolic and autoimmune diseases or tumor progression, the discipline has paved the way for harnessing therapeutic cell implants in vivo^3^. Such devices can also detect the onset of microbial infections by scoring pathogenic markers released from damaged tissue^4^. Synthetic–biological approaches have also been utilized in the discovery of antimicrobials^5^. However, bacterial drug resistance is becoming an increasing global health concern, and new treatment alternatives to antibiotics, that reduce bacterial virulence without applying selective pressure on essential pathways are urgently needed^6^.

An attractive strategy for this purpose is to target non-essential bacterial communication via diffusible signaling molecules called autoinducers, which synchronize collective bacterial behavior^7^. The increase of autoinducer levels with increasing bacterial population density orchestrates population-wide gene expression through a process called quorum sensing^8^. This enables unicellular bacteria to function as a multicellular-like organism, and synchronizes behaviors, such as pathogenicity, motility, biofilm formation, and antibiotic resistance^9,10^. Many prokaryotes have evolved signals for interspecies or host interactions^11^, but the family of interconverting autoinducer-2 (AI-2) molecules derived from 4,5-dihydroxy-2,3-pentanedione appears almost ubiquitously as a communication signaling system throughout the bacterial kingdom. AI-2 has been shown to promote the treatment of *Vibrio cholerae* infections^10^ and microbial biofilms^12,13^, and also helps in reestablishing a healthy gut microbiome by favoring the expansion of *Firmicutes* over *Bacteroidetes*^14^ populations even in the presence of commensal AI-2 secreting bacteria without imposing drastic selection pressure^6^. Therefore, in order to develop a synthetic biology-based device to mediate cell-based anti-infective activity, we selected AI-2 as the effector component. However, it is important to keep in mind that in other bacteria AI-2 can also have consequences that can promote virulence, rather than attenuating it, such as autoaggregation of *Escherichia coli*^15^ and the release of phage by *Enterococcus faecalis*^16^. Thus, this aspect needs to be considered when targeting quorum sensing.

On the other hand, for the detection of infection, we focused on cell-surface formyl peptide receptors (FPR)^17^ that detect invading pathogens releasing formyl peptides such as N-formylmethionyl-leucyl phenylalanine (fMLF). Only the most potent amongst the three members of the human FPR receptor family, FPR1, monitors the presence of nanomolar concentrations of chemotactic N-formylated peptides^18^, and is therefore suitable for early-stage detection of infections. Formylated peptides are ubiquitous by-products of prokaryotic protein translation released by invading microorganisms, and progressively guide phagocytes to the site of infection or inflammation^19^. Formylated peptides serve as signal peptides in bacteria and are released after translocation of the carrier protein across the cytoplasmic membrane^17^. Over 300 bacterial species have been identified to produce formylated peptides with variable sequence and length which activate human FPR1 at low nanomolar concentrations^17^. The prototypic tripeptide fMLF^20^ produced by opportunistic *E. coli* and the tetrapeptide N-formyl-Met-Ile-Phe-Leu (fMIFL) released by *Staphylococcus aureus*^21^ are among the most potent FPR1 agonists know today. While N-terminal fMet most likely acts as protein degradation signal in bacteria^22^, it has not been reported in unicellular eukaryotes or in higher organisms outside mitochondria or damaged tissue^19^.

Because native chemotaxis-associated G-protein-coupled receptors expressed by human neutrophils^23^ specifically interact with diffusible pathogen-derived formylated peptides, we hypothesized that this biosensor/biomarker combination could be engineered to interface with a broad range of potential infections. Further, we considered that a synthetic formyl peptide sensor (FPS) module could be coupled with an AI-2 release module that would act upon infections by autonomously reprogramming bacterial communication. The presence of any bacteria in the bloodstream represents an acute life-threatening situation (e.g., sepsis), which may be attenuated by the secretion of quorum-sensing molecules. Implanted designer cells monitoring the presence of bacteria via circulating biomarkers such as formyl peptides ensure that AI-2 will be released at the right time and place. Like for other synthetic biology-inspired designer cell-based proof-of-concept therapies^24^, the real therapeutic setting will be based on subcutaneous implantation of encapsulated designer cells. Encapsulation protects the designer cells from the host immune system as well as the host from the designer cells for months^25^ and enables continuous oxygen supply by promoting autovascularization^25,26^, the connection of the designer cells to the bloodstream of the patient.

Based on the above ideas, we report here the design, development and evaluation of a synthetic biology-inspired gene circuit, which we call microbial-control device, that bridges the interface gap between mammalian cells and microbes by utilizing cell-derived quorum-sensing molecules to autonomously regulate microbial behaviors, and in some bacteria, inhibit virulence. Essentially, host cells can be programmed to control infectious organisms. When challenged with autoinducer-responsive pathogens, microbial-control-engineered cells detect microbial peptides with high sensitivity and secrete AI-2 to control quorum-sensing-related behavior and reduce microbial biofilm formation of several clinically relevant species. Given the plethora of bacterial species endowed with AI-2 signaling systems, we believe this microbial-control device represents a promising prototype of cell-based anti-infective systems that mimic host-microbe cross-talk.

## Results

### Design of a synthetic mammalian microbial-control device

**Fig. 1:**
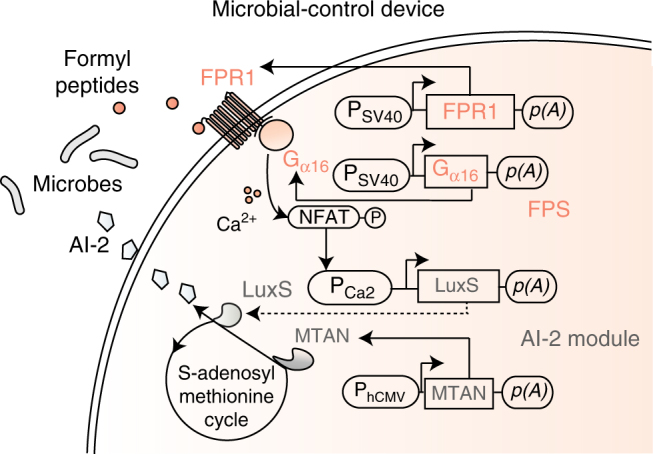
Design of the cross-kingdom microbial-control device. A synthetic gene network constantly monitors the presence of pathogen-derived formyl peptides and programs the corresponding production of autoinducer-2 (AI-2) by human HEK-293 cells. In particular, (i) N-formyl peptides activate the sensor module consisting of ectopically expressed human formyl peptide receptor 1 (FPR1; P_SV40_-FPR1-pA, pFS98) rewired to the constitutively expressed promiscuous human G protein subunit Gα_16_ (P_SV40_-Gα_16_-pA, pFS102) and relays to calcium-triggered expression of S-ribosylhomocysteinase (LuxS; P_Ca2_-luxS-pA, pFS186). (ii) The ectopically expressed methylthioadenosine nucleosidase (MTAN, P_hCMV_-MTAN-pA, pFS84) supplies the terminal AI-2 catalytic component S-ribosylhomocysteine lyase (LuxS) with the artificial methionine cycle intermediate S-ribosylhomocysteine to generate AI-2) (iii) AI-2-responsive pathogens detect the secreted quorum-sensing signal released from the microbial-control cells and adapt quorum-sensing-controlled behavior

The synthetic microbial-control device with programmable AI-2 release was designed as a formyl peptide-responsive signaling cascade with a downstream AI-2 biosynthesis module (Fig. 1). The formylated peptide sensor (FPS) module consists of a constitutively expressed formyl peptide receptor 1 (FPR1), sensitive to N-formylated peptides of microbial origin, coupled with a promiscuous G protein that confers the artificial ability to stimulate calcium signaling onto compatible G_αi_-coupled receptors^27^. Ectopic expression of the human membrane-bound G protein-coupled receptor FPR1 was therefore genetically rewired to the ectopically expressed human G protein subunit G_α16_ that reroutes the G_αi_-coupled FPR1 towards Ca^2+^ signaling^28^. Once activated through FPR1, the G_α16_ initiates intracellular Ca^2+^ signaling via phospholipase C-β and enables the transcription factor nuclear factor of activated T cells (NFAT) to bind and activate synthetic P_NFAT_-derived promoters^24^. Compatible with the downstream signaling of the sensor unit, the AI-2 production platform involves ectopic expression of two biosynthetic enzymes optimized for mammalian expression, namely methylthioadenosine/S^−^adenosylhomocysteine nucleosidase (MTAN) and S-ribosylhomocysteine lyase (LuxS), which serve to sequentially produce and secrete the universal microbial communication signal AI^−^2^29,30^. Constitutively expressed *mtaN* yields the intermediate metabolite S^−^ribosylhomocysteine (SRH), while *luxS* expression, either constitutive or under control of a synthetic P_Ca2_ promoter, guides the conversion of the SRH intermediate into a set of interconverting AI-2 signaling molecules^31,32^. Rewiring of the FPS^−^derived signaling cascade to the Ca^2+^-driven synthetic promoter links both modules, completing the microbial-control circuit.

### Validation of the pathogen-detecting FPS

**Fig. 2:**
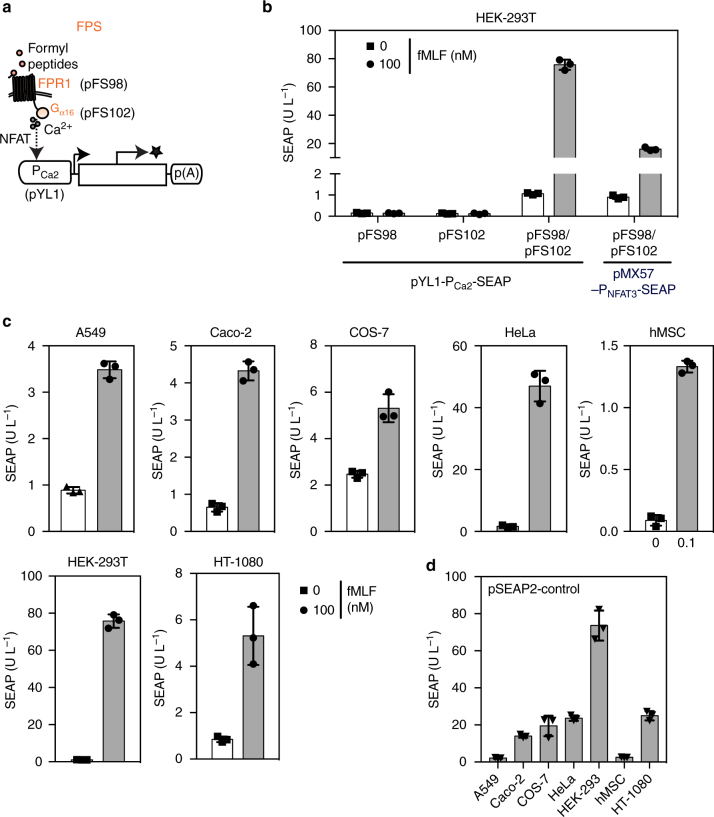
A formyl peptide-sensitive transcription control module. **a** Schematic representation of the formyl peptide sensor (FPS) connected to intracellular (Citrine; P_Ca2_-Citrine-pA, pFS220) or secreted (SEAP; P_Ca2_-SEAP-pA, pYL1) reporter proteins. **b** Validation of FPS components. HEK-293 cells transgenic for the FPS were cotransfected with the G protein-coupled receptor FPR1 (P_SV40_-FPR1-pA, pFS98), the G Protein G_α16_ (P_SV40_-G_α16_-pA, pFS102) and a calcium-responsive reporter, (P_Ca2_-SEAP-pA, pYL1) or (P_NFAT3_-SEAP-pA, pMX57), to produce SEAP in response to fMLF, in contrast to control cells lacking ​either the calcium pathway-rerouting Gα_16_ or the FPR1 sensory module. **c** Formyl peptide-induced SEAP expression in different cell lines. HeLa, COS-7 and HEK-293 were cotransfected with the FPR1-encoding expression vector (P_SV40_-FPR1-pA, pFS98), the G_α16_-encoding expression vector (P_SV40_-G_α16_-pA, pFS102) and the P_Ca2_-driven SEAP reporter plasmid (P_Ca2_-SEAP-pA, pYL1), while hMSC-TERT, A549, Caco-2 and HT-1080 were engineered with the FPR1-encoding expression vector featuring the human elongation factor 1 alpha (P_EFIα_) promoter (P_EFIα_-FPR1-pA, pFS115), the P_hCMV_-driven Gα_16_ expression vector (P_hCMV_-Gα_16_-pA_,_ pcDNA3.1-G_α16_), together with the reporter plasmid (pYL1). Cells were grown in the presence or absence of N-formyl peptide, and SEAP levels were profiled in the culture supernatant after 24 h. **d** Transfection efficiency of engineered cell lines. Cells were transfected with a constitutive SEAP expression vector (pSEAP2-control) and SEAP levels were quantified after 24 h. Data are means ± SD and symbols indicate means of individual experiments (*n* = 3)

We initially established and optimized the components for the sensor unit (FPS). For this purpose, we connected constitutively expressed FPR1 receptors (P_SV40_/P_hEF1α_-FPR1-pA, pFS98/pFS115) to the G_α16_ adapter protein (P_hCMV_/P_SV40_-Gα16-pA; pcDNA3.1-Gα16/pFS102) under control of fine-tuned promoter strengths and in a defined ratio, and linked them to Ca^2+^-dependent expression of SEAP (human placental secreted alkaline phosphatase; P_Ca2_-SEAP-pA, pYL1) or cytosolic Citrine, an enhanced YFP (P_Ca2_-Citrine-pA, pFS220) reporter (Fig. 2a). Cotransfection of HEK-293 cells with all three components of the FPS network (pFS98/pFS102/pYL1) validated formyl peptide-dependent target gene expression (Fig. 2b). FPS-activating fMLF levels are in the physiologically relevant concentration range, since fMLF levels over 50 nM efficiently stimulate FPR1 or the innate immune response^33^.

As the reporter configuration featuring a combination of CRE, SRE and NFAT response elements upstream of a minimal TATA-box promoter (pYL1) provided a superior induction profile and higher maximum formyl peptide-dependent expression levels than simple P_NFAT_-driven promoters (pMX57), we used the former as the preferred reporter scaffold for follow-up experiments. The FPS module demonstrated functionality in different FPR1/G_α16_-cotransfected mammalian cell lines such as human mesenchymal stem cells (hMSC-TERT), human alveolar epithelial cells (A549) and colorectal epithelial cells (Caco-2) (Fig. 2c), suggesting broad applicability of the synthetic bacterial detection cascade with altered induction profiles among different cell lines^34^. Variations in the overall performance of the FPS module in different cell types reflects the combinatorial impact of endogenous Gα levels, ectopic FPR1 expression^35^, the NFAT transcription factor repertoire, transfection efficiency (Fig. 2d), and the protein secretion capacity of the specific cell types. Stronger promoters driving higher FPR1 and Gα16 expression levels increased the performance of the FPS module in Caco-2 cells (Supplementary Fig. 1a) and hMSCs (Supplementary Fig. 1b).

Additionally, fine-tuning of the relative FPR1/G_α16_ expression levels substantially increased FPS performance (Supplementary Fig. 1c), in particular when G_α16_ levels exceeded those of other G-proteins such as G_αi2_ (Supplementary Fig. 1d). Since HEK-293 showed the best microbial peptide-triggered expression performance, this cell line was chosen for subsequent experiments.

**Fig. 3:**
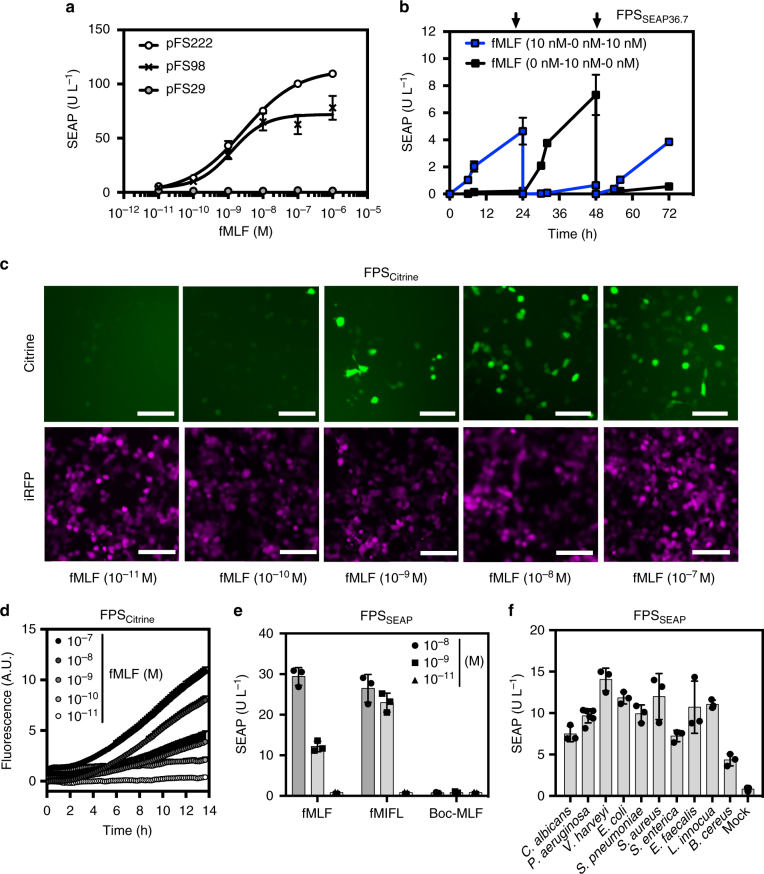
Characterization of the formyl peptide transgene switch. **a** Formylated peptide sensitivity and adjustability of the FPS in HEK-293 cells cotransfected with pFS102 (P_SV40_-G_α16_-pA) plus pYL1 (P_Ca2_-SEAP-pA) and either pFS98 (P_SV40_-FPR1-pA), pFS222 (P_SV40_-FPR1ΔST-pA) or with pFS29 (P_SV40_-mCherry-pA). SEAP expression was quantified after 24 h. The data points were fitted using a sigmoidal dose-response curve. **b** Reversibility of formyl peptide-responsive SEAP expression assessed by cultivating stable FPS_SEAP36.7_ cells for 72 h while alternating the fMLF levels (0/10 nM) every 24 h and recurrent fMLF exposure for 1 h. Arrows indicate medium change. **c** Fluorescence micrographs of FPS_Citrine_ (pFS98/pFS102/pFS220) and iRFP670 (P_hCMV_-iRFP670-pA, pMM581)-cotransfected cell populations cultivated for 24 h in the presence of different concentrations of fMLF. Scale bar = 100 μm. **d** Real-time monitoring of formyl peptide-triggered cellular fluorescence of FPS_Citrine_-engineered cell populations (pFS98/pFS102/pFS220) by time-lapse fluorescence microscopy. **e** FPS specificity towards N-terminal modified peptides. Engineered FPS_SEAP_ cell populations were cultivated for 24 h in the presence of prototypic types of FPR1 agonists (fMIFL and fMLF) or a synthetic FPR1 antagonist (Boc-MLF) before SEAP expression was assessed in the cell culture supernatant. **f** Formyl peptide-triggered responses to supernatants of opportunistic pathogens of FPS_SEAP_-engineered cell populations after 24 h. Data show the means ± SD of three experiments

Further characterization of the FPS in HEK-293 cell populations demonstrated that the sensor circuit featuring the human FPR1 receptor (pFS98/pFS102) robustly responded with low nanomolar sensitivity (EC_50_ = 2 nM) to a wide range of formyl peptide concentrations, while a receptor variant engineered for decreased internalization and desensitization^36^ (FPR1ΔST; P_SV40_-FPR1ΔST-pA/pFS222) displayed a comparable dose-response profile (EC_50_ = 4 nM) combined with a broader dynamic range (Fig. 3a). As the FPS configuration featuring FPR1 (pFS98) demonstrated the highest sensitivity based on its effective concentration (EC_50_) in HEK-293 combined with best fold induction in the nanomolar fMLF range, this receptor was selected for follow-up experiments.

To assess whether stable chromosomal integration of the FPS components (Supplementary Fig. 2a) into HEK-293 cells would allow long-term stability and responsiveness of the FPS network, we selected stable FPS_SEAP36_ monoclonal cell populations and profiled reporter gene expression after induction with low amounts of fMLF (10 nM) to favor the identification of highly sensitive clones (Supplementary Fig. 2b). The monoclonal cell population FPS_SEAP36.7_ (FPS_SEAP36_ clone 7) was the best-performing formyl peptide-responsive transgenic cell line showing high sensitivity (EC_50_ = 1 nM) and SEAP induction factor (9-fold; Supplementary Fig. 2c), full reversibility characterized by sequential ON/OFF switches following addition and withdrawal of formylated peptides (Fig. 3b) as well as long-term sensitivity^24^ to microbial formylated peptides for a testing period of over 3 weeks (Supplementary Fig. 2d). High sensitivity of the FPS to the microbial chemoattractant fMLF and a rapid output profile were confirmed by real-time monitoring of the intracellular fluorescent reporter Citrine in pFS98/pFS102/pFS220-co-transfected (FPS_Citrine_) cell populations (Fig. 3c). These results indicated that the FPS delivers a rapid response within 3 h after detection of formylated peptides (Fig. 3d), which is considered a fast transcription response^37,38^ and would allow the detection of pathogens at an early phase of infection.

In addition to *E. coli*-derived fMLF, FPS_SEAP_-engineered cells also reliably recognized the N-formyl peptide fMIFL, a prototypic peptide released from *S. aureus*, while the corresponding non-formylated tripeptide t-butyloxycarbonyl-MLF (BocMLF), a competitive FPR1 antagonist^39^, failed to activate FPR1^14^ (Fig. 3e). Having demonstrated selectivity and functionality of the FPS upon contact with microbial peptides, we next tested its ability to trace various pathogens. To this end, transient FPS_SEAP_-transgenic designer cells, engineered to produce SEAP in response to formyl peptides, were challenged with culture supernatants from different human pathogens and model organisms, such as the opportunistic pathogens *Bacillus cereus*, *Vibrio harveyi, E. coli, Listeria innocua*^40^ and *Candida albicans*^41^ or the notoriously difficult-to-treat nosocomial pathogens *Pseudomonas aeruginosa*^42^, *S. aureus Streptococcus pneumoniae*, *Salmonella enterica and Enterococcus faecalis* (Fig. 3f).

We observed that SEAP levels were exclusively increased by supernatants obtained from prokaryotes and *C. albicans*. In contrast *Saccharomyces cerevisiae*, which do not produce formylated peptides^41^ and therefore served as negative control, did not induce SEAP expression (Supplementary Fig. 3a**)**, which implies sensitivity of the FPS to N-formyl peptides at the concentrations found in nature (Supplementary Table 2). Bacterial cell densities used to produce the formylated peptide-containing culture supernatants (10^6^ CFUs/ml) are similar to those reported for in vivo conditions^43,44^. *E. coli* culture supernatants activated the FPS system to a high level since *E. coli-*derived fMLF is one of the most potent FPR1 agonists^20^ (Fig. 3f). HEK-293’s endogenous G_αq_-coupled receptors that also signal through the Ca^2+^-dependent pathway did not interfere with the FPS module at physiological norepinephrine and acetylcholine concentrations (Supplementary Fig. 3b), confirming previous results with other important signaling pathways^38^. Additionally, the FPS module was neither substantially activated by mitochondria-derived formylated peptides released by cell debris containing (Supplementary Fig. 3c) nor by human serum (Supplementary Fig. 3d). Control experiments showed that the cell viability was not affected by the presence of formyl peptides (Supplementary Fig. 3e, f).

### A mammalian AI-2 designer biosynthesis pathway

**Fig. 4:**
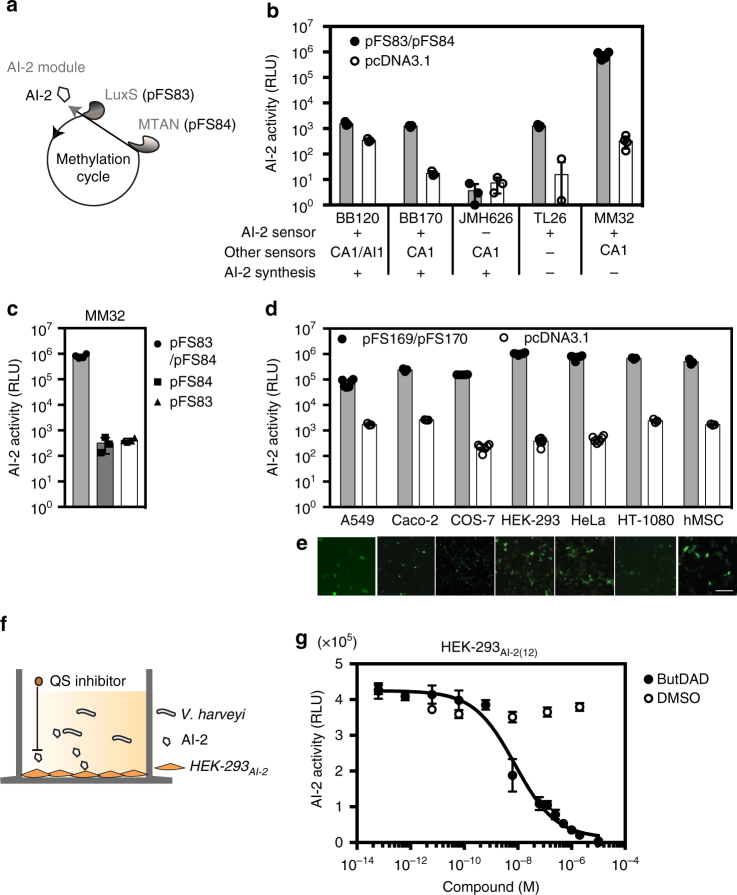
Validation of artificial human autoinducer-2 synthesis. **a** Schematic representation of the methylation cycle by-pass for continuous AI-2 biosynthesis. **b** Bioluminescence of *V. harveyi* strains showing distinct sensor properties in response to supernatants of AI-2 module-engineered HEK-293 cells. Cells co-transfected with LuxS (P_hCMV_-LuxS-pA, pFS83) and MTAN (P_hCMV_-MTAN-pA, pFS84) or pcDNA3.1-transfected were cultivated for 24 h before culture supernatants were tested for AI-2. **c** *V. harveyi* (MM32) bioluminescence triggered by supernatants from HEK-293 expressing both or single AI-2 biosynthetic enzymes. **d** AI-2 secretion in different cell lines. Mammalian cells cotransfected with expression vectors coding for MTAN-eYFP (P_hCMV_-*mtaN*-*eyfp*-pA, pFS170) and LuxS-eYFP (P_hCMV_-*luxS*-*eyfp*-pA, pFS169) prior to measuring AI-2 activity from the culture supernatant 24 h later. **e** Representative micrographs of fluorescent AI-2 producing cell lines transgenic for MTAN-eYFP (pFS170) and LuxS-eYFP (pFS169) visualized by fluorescence microscopy after 24 h. Scale bar = 100 μm. **f** Illustration of cell-based quorum-sensing inhibitor evaluation by challenging engineered LuxS-/MTAN-transgenic HEK-293_AI-2_ cells with an inhibitor compound followed by co-cultivation with *V. harveyi* (MM32). **g** Monitoring of AI-2 synthesis inhibition of HEK-293_AI-2(12)_ by BuT-DADMe-Immunicillin-A (ButDAD). HEK-293_AI-2(12)_ were exposed for 24 h to different compounds and co-cultured with *V. harveyi* (MM32) for 6 h before recording bioluminescence. Data show the means ± SD of triplicate experiments (*n* = 3)

To automatically link the onset of infection to an output that modulates or counteracts microbial behavior, we then implemented an AI-2 production platform seamlessly integrating with human cellular metabolism. The AI-2 unit harbors a synthetic metabolic pathway that produces and secretes the common quorum-sensing molecule AI-2 within mammalian cells (Fig. 4a). Based on the well-described *E. coli* AI-2 biosynthesis pathway^31^, we speculated that ectopic overexpression of human codon-optimized *mtaN* would partially by-pass the human endogenous methylation cycle (Supplementary Fig. 4a) by converting the waste product S-adenosylhomocysteine (SAH) into SRH. Preventing SRH accumulation by additional ectopic expression of human-codon-optimized AI-2 synthase (*luxS*) should lead to production of AI-2 via 4,5-dihydroxypentane-2,3-dione (DPD) as an intermediate^45^. Indeed, HEK-293 cells co-transfected with the *E. coli*-derived enzymes MTAN (P_hCMV_-MTAN-pA, pFS84) and LuxS (P_hCMV_-LuxS-pA, pFS83) extracellularly accumulated bioactive AI-2. Heterologous AI-2 production was profiled by various specific *V. harveyi* AI-2 reporter strains, including the two most reliable AI-2 *V. harveyi* sensor strains that presently exist, BB170 and MM32. BB170 harbors a knockout in the AI-1 sensor to prevent interference by the unrelated AI-1 and MM32 contains an additional AI-2 production deficiency. The control strain JMH626 (sensitive only to the *Vibrio cholerae* autoinducer CAI-1) excluded the possibility of unspecific AI-2 reporter activation (Fig. 4b)^46^. The two-component AI-2 production platform thus provides efficient autoinducer synthesis in pFS83/pFS84-co-transfected HEK-293 cells (Fig. 4b), in sharp contrast to cells harboring only the individual components (Fig. 4c).

Mammalian cells are very good heterologous protein producers, hence their importance for the production of bioparmaceuticals, but are very poor small-molecule producers^47^. Therefore, we have tested a variety of standard mammalian cell lines (HeLa, hMSC, HT-1080), including cell types that are targets for bacterial infections (A549, Caco-2), to evaluate the best-in-class small-molecule producer that can synthesize AI-2 at the highest possible level. AI-2 secretion from different mammalian cell lines co-transfected with constitutive AI-2 expression vectors (P_hCMV_-LuxS-eYFP-pA, pFS169) and MTAN-eYFP (P_hCMV_-MTAN-eYFP-pA, pFS170) confirmed that HEK-293 cells, which already exhibited the best FPS induction profile, also efficiently secreted AI-2 into the culture medium (Fig. 4d). Expression of fluorescent AI-2 converting enzymes was observed in all AI-2 positive cell lines (Fig. 4e). The overall AI-2 production level results from a combination of (i) transfection efficiency of plasmids encoding the AI-2-biosynthetic enzymes, (ii) the expression levels of AI-2-biosynthetic enzymes, (iii) activity and metabolic impact of AI-2-synthesizing enzymes in the heterologous mammalian host, (iv) availability of the substrate SAH and cofactors in the mammalian cytosol and (v) the secretion of the heterologous AI-2 through the plasma membrane.

Neither metabolic engineering with AI-2-biosynthetic enzymes (Supplementary Fig. 4b) nor the addition of AI-2 to cultured cells (Supplementary Fig. 4c) did affect the overall cell viability. Ectopic expression of intracellular AI-2-biosynthetic enzymes (Supplementary Fig. 4d) did not impact cell morphology (Supplementary Fig. 4e). AI-2 activity in (pFS83/pFS84)-transgenic HEK-293 cells was already detectable within 30 min in culture supernatants based on *V. harveyi* (BB170) bioluminescence (Supplementary Fig. 5a), and after 24 h cellular AI-2 release reached micromolar levels (2 µM) (Supplementary Fig. 5b), which lies within the lower autoinducer concentration range commonly found in bacteria such as *E. coli*^48^ during logarithmic growth (Supplementary Fig. 5c). A selected stable HEK-293_AI-2_ monoclonal cell population with stable chromosomal integration of both *mtaN* and *luxS-eyfp* (Supplementary Fig. 6a), combining efficient AI-2 secretion from the previously characterized transient AI-2 module and the highest production level of AI-2, termed HEK-293_AI-2(12)_ (clone number 12), was used in further experiments (Supplementary Fig. 6b).

### Cell-based validation of quorum-sensing inhibitors

We further aimed to harness cellular AI-2 production from the monoclonal population to evaluate AI-2 biosynthesis inhibitors in a cell-based assay; previously, only bacterial or biochemical assays have been available for this purpose^49^. In principle, human cell-based assays provide much more information than simple mechanistic MTAN inhibition which is highly relevant for further drug development:^5^ (i) Non-cytotoxicity of the compound to human cells, (ii) bioavailability of compounds such as diffusion into human cells to target intracellular pathogens and (iii) the absence of off-target effects towards the human MTA phosphorylase^50^. The cell-based assay for detection of AI-2 biosynthesis inhibitors targeting LuxS or MTAN combines the HEK-293_AI-2(12)_ AI-2 module with the AI-2 sensing abilities of *V. harveyi* (LuxS^-^, MM32) in a single well of a high-throughput-compatible 96-well plate (Fig. 4f). We tested several rationally designed transition state analogs of AI-2 biosynthetic enzymes that showed the strongest inhibition of LuxS^51^ or MTAN in vitro, including a compound that outperforms natural AI-2 synthesis inhibitors^52^. Challenging HEK-293_AI-2(12)_ with the first rationally designed compound targeting MTAN revealed that But-DADMe-ImmA^53^ is a specific inhibitor with an IC_50_ value of 7.7 nM (Fig. 4g). This cell-permeable compound was bioavailable, non-cytotoxic (Supplementary Fig. 7a, b) while it substantially reduced quorum-sensing-associated characteristics of *V. harveyi* without compromising the pathogen’s capability to detect AI-2 (Supplementary Fig. 7c). The assay could be further extended to evaluate a small set of rationally-designed LuxS inhibitors among which SK2-93 (IC_50_ = 3.4 µM)^51^ (Supplementary Fig. 8a**)** showed the best characteristics followed by SK2-92 (IC_50_ = 73 µM)^54^ (Supplementary Fig. 8b). Targeting either LuxS, MTAN or both building blocks of the AI-2 module resulted in AI-2 release with NOR logic gate behavior and hence validated sequential activity of the AI-2 biosynthesis components (Supplementary Fig. 8c**)**.

### Programming quorum-sensing interference with human pathogens

**Fig. 5:**
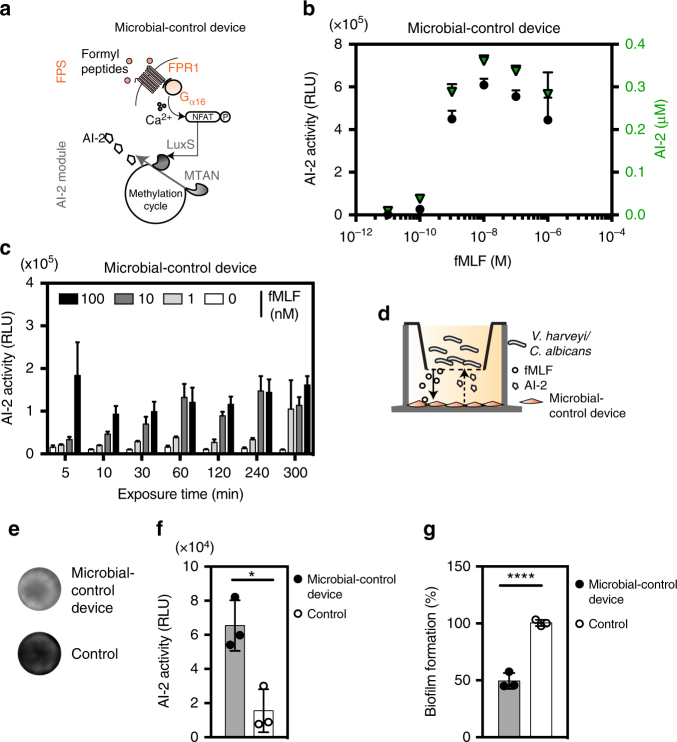
Validation of the fMLF-responsive microbial-control device. **a** Illustration of tunable AI-2 release from the microbial-control device. **b** Microbial-control-transgenic pFS98/pFS102/pFS84/pFS186-cotransfected HEK-293 cells were grown for 24 h in cell culture medium adjusted to increasing fMLF levels, before AI-2 activities were profiled (black) and AI-2 concentrations were determined (green) in the culture supernatant. **c** Impact of fMLF doses and induction time on microbial-control-mediated AI-2 secretion. AI-2 release profile from microbial-control cells exposed to rising fMLF levels and induction pulses after 24 h. AI-2 activity was measured in terms of *V. harveyi* (MM32) bioluminescence. **d** Autonomous control of microbial pathogens through microbial-control-engineered cells. Schematic depicts the release of formyl peptides from microbes. Diffusible formyl peptides activate microbial-control cells to release AI-2, which, in turn, induces bacterial bioluminescence or reduces biofilm formation. **e**, **f** Microbial-control or mock-engineered HEK-293 cells (Control) were seeded in the lower chamber of a Transwell, and then co-cultivated with *V. harveyi* AI-2 reporter strain (MM32, *luxS*^*-*^) in the upper compartment. Twenty hours after incubation with mammalian cells, *V. harveyi* bioluminescence was **e** imaged and **f** quantified. **g** HEK-293 co-transfected with the microbial-control device components or transfected with pcDNA3.1 (Control) were grown in the bottom chamber and then challenged with *C. albicans* in the upper compartment. After co-cultivation for 24 h, *C. albicans* biofilm formation was measured by means of MTT assay. Biofilm formation is expressed as a percentage of the control value of *C. albicans* co-cultivated with HEK-293 control cells lacking the genetic components. Data represent the means ± SD of three independent experiments measured in triplicate. *p < 0.05, ****p < 0.00001, two-tailed Student’s *t*-test

After validation and optimization of the first module for high formyl peptide sensitivity and the second module for maximum AI-2 secretion in human and primate cells, we assembled the microbial-control circuit by docking the FPS to the AI-2 module. Cotransfection of HEK-293 cells with the FPS components (pFS98/pFS102) in combination with a Ca^2+^-dependent version of the AI-2 module harboring P_Ca2_-*luxS*-pA (pFS186) linked to constitutively expressed *mtaN* (pFS84) afforded programmable microbial-control cell populations (Fig. 5a). We confirmed that cells engineered with the microbial-control device (pFS98/pFS102/pFS84/pFS186) detected nanomolar levels of N-formyl peptide and responded by dynamically adjusting cellular AI-2 release in the sub-micromolar range, as shown by up to 330-fold bioluminescence induction of *V. harveyi* (Fig. 5b).

AI-2 predominantly impacts bacteria during their logarithmic growth. Therefore, we also confirmed dynamic AI-2 release in response to increasing induction pulse durations at different physiological fMLF concentrations: high formyl peptide levels quickly turned on AI-2 production, whereas lower levels required a longer induction pulse period to generate significant AI-2 activity in the extracellular environment (Fig. 5c). The microbial-control device was also programmable inside a variety of human cells, confirming the broad applicability of the system (Supplementary Fig. 9a). Although fMLF-triggered AI-2 production may appear moderate in some cell types, the absolute AI-2 levels produced by all cell types are within the clinically effective range^55,56^. Since HEK-293 showed the best overall fMLF-mediated control performance they were used as parental cell type for all follow-up experiments. Currently, HEK-293 cells emerge as the optimal parental cell line for the design of synthetic biology-based circuits because of their increased production capacity, transfection rate and cultivation robustness^24,26^. We confirmed this notion by generating monoclonal penta-transgenic HEK-293-derived FPS_AI2_ cell populations (Supplementary Fig. 9b) whose fMLF-triggered AI-2 production was profiled over a wide fMLF concentration range (Supplementary Fig. 9c) and remained robust for a testing period of over 3 weeks (Supplementary Fig. 9d).

To verify the functionality of the microbial-control device and to assess whether the microbial-control two-way communication system indeed modifies quorum-sensing-controlled pathogenic behavior in an infection-like environment, we first profiled bacterial quorum-sensing-related behavior in the presence of microbial-control-engineered cells. To demonstrate remote control of collective bacterial behavior, we chose the AI-2-deficient quorum-sensing variant of the model organism *V. harveyi* (*luxS*^-^*)* and a co-incubation model featuring Transwell permeable supports, which allows bacterial formyl peptides and signaling molecules such as AI-2 (or any secretable molecules) from mammalian cells to pass. The small membrane pore size (0.3 µm) allows HEK-293-derived microbial-control cells to communicate with bacteria, while shielding them from direct pathogen contact (Fig. 5d). Formyl peptides released from *V. harveyi* as an indicator for bacterial density were detected by the FPS sensory module of microbial-control-engineered (pFS98/pFS102/pFS84/pFS186) HEK-293 cells. Designer cells subsequently initiated AI-2 biosynthesis and secretion, directly addressed responsive *V. harveyi* in the upper compartment and orchestrated bacterial luminescence. The increased *V. harveyi* bioluminescence confirmed AI-2-mediated inter-species quorum sensing (Fig. 5e, f).

Finally, the microbial-control device from engineered HEK-293 cells was applied to the opportunistic human fungal pathogen *C. albicans* to test its ability to inhibit biofilm formation, since AI-2 has been reported to inhibit *C. albicans* biofilm formation in a dose-dependent manner^9^. We used a Transwell setup with a porous filter membrane to separate pathogenic yeast in the upper compartment from adherent microbial-control cells in the bottom compartment while permitting free molecule diffusion (Fig. 5d). Experimental analysis of microbial-control cells challenged with *C. albicans* confirmed that biofilm formation of co-cultured *C. albicans* was substantially reduced by half within 24 h, while control cells failed to inhibit biofilm formation (Fig. 5g).

AI-2’s prophylactic impact on the prevention of microbial biofilms^13^ could also be confirmed in follow-up experiments using the opportunistic human pathogen *B. cereus* (Supplementary Fig. 10a) and in mixed species communities containing *B. cereus* as well as *E. coli* (Supplementary Fig. 10b).

AI-2-dependent reduction of *C. albicans*’ biofilm formation was most significant during early growth phase (Supplementary Fig. 10c**)**, while AI-2 interventions at higher cell densities had only a modest effect on biofilm formation (Supplementary Fig. 10d**)**. Decreased biofilm formation observed for *C. albicans* correlated with increased AI-2 secretion from microbial-control engineered cells following exposure to the pathogenic yeast (Supplementary Fig. 10e). Control experiments exploring the impact of AI-2-secreting cells on the composition of mixed-species communities^14^ did not reveal significant changes (Supplementary Fig. 10f), which is in line with the fact that AI-2 does not shift compositions of native commensal communities in the absence of antibiotics^14^.

Thus, the microbial-control device robustly detected pathogens, coordinated AI-2 release exclusively in response to microbial peptides, and prevented fungal biofilm formation.

## Discussion

Synthetic biology enables cell responses to be tailored to a diverse combinatorial repertoire of input signals ranging from small molecules to peptides^57^, but technology for specific detection of microbial invaders has so far only been implemented in prokaryotes^42,58^. We considered that a two-way communication interface between designer cells and microbes might be applied to reprogram bacterial quorum-sensing-controlled traits with certain precautions, providing an alternative approach to address the issue of microbial resistance to antibiotics. Here, we describe the assembly, characterization and validation of designer cells containing an microbial-control device that senses the presence of microbial peptides and exploits the autonomously produced communication signal AI-2 to influence responsive bacteria and human pathogenic yeast. This cell-based microbial control principle represents a mammalian cell-bacteria communication system and mimics features of host-microbial symbioses vital to human health, such as two-way communication and mutual feedback^59^. Its design was inspired by the recently discovered, though still poorly understood, host-microbe crosstalk that permits pathogens to escape traditional cellular defense mechanisms^11,60^. Formylated peptides appear to be a consistent indicator of bacterial infections and hence can serve as an efficient input molecule, which is why the microbial-control device is designed to exclusively detect formylated peptides as a proxy for the presence of microbes in the bloodstream and will release non-toxic AI-2 exclusively into the bloodstream. Depending on the amount of formylated peptides present, the AI-2 output level can be fine-tuned to the therapeutically effective range matching the physiological AI-2 levels produced by bacteria^61^ communicating via quorum sensing^9,56^. The engineered interface expands the known inter-kingdom vocabulary consisting of mammalian stress hormones and synthetic inducers^62,63^ to the fully active AI-2 communication signal, and is expected to have the ability to drive global changes in gene expression in bacteria causing systemic infections, but could further impact mixed microbial populations such as those that exist in the gut or the oral cavity^8^.

In regards to multiresistent or chronic infection, a prophylactic cellular implant might remotely monitor and fight the source of infection without establishing the selective pressure resulting in the emergence of drug resistance in current antibiotic treatment schemes^6,64^. To drive designer cell-based anti-infective implants into a clinical reality, the designer cells need to be implanted inside semi-permeable, autovascularizing and immunoprotective microcontainers,^25,38^. The designer cells will detect the presence of invading microbes via formylated peptides in the circulation and coordinate expression of native factors that take control over bacterial group behavior in the absence of any selective pressure. With the dramatically increasing prevalence of multidrug-resistant human-pathogenic bacteria new anti-infective treatment strategies are urgently needed^42,65^. The synthetic biology inspired microbial-control device could kick-off new opportunities in future anti-infective therapies. This strategy holds great promise for future clinical applications of the microbial-control cells, for it has already been tested in a human clinical trial^25^.

Importantly, our findings indicate that mammalian cells can synthesize bioactive furanosyl borate diester and other members of the AI-2 family of signaling molecules, and are resistant to the metabolic burden of the by-passed methylation cycle. Thus, there should be opportunities to extend designer cell implants towards the biosynthesis of other secretable small molecules.

Due to the inherent flexibility of both the receptor and the chosen species-independent AI-2 output element, diverse pathogens can be targeted, ranging from prokaryotes to fungi^41^. The remarkable sensitivity of the sensor circuit (0.1 nM) derives from the strong interaction of formyl peptides with their cognate receptor and is compatible with the systemic concentrations of a myriad of closely related formyl peptides upon infection. Once activated, the synthetic sensor cascade dynamically triggers the expression of AI-2 biosynthetic enzymes according to the levels of formylated peptides present, amplifies the input signal and provides strong and long-lasting enzymatic autoinducer release. Also, AI-2 is not expected to be toxic at the levels secreted by the designer cells, since AI-2 exposure is well tolerated by human cells^66^.

The microbial-control device could further be used for (i) AI-2 release from implanted microencapsulated designer cells, (ii) to rapidly redirect bacterial motility and hence, (iii) to precisely target bacterial therapeutics in vivo through AI‐2‐mediated chemotaxis^3,67^, or (iv) for cell-based AI-2 inhibitor screening. Cell-based assays can identify noncytotoxic and bioavailable compounds accessing the microbial habitat of quorum-sensing pathogens and have already enabled the discovery of anti-infective compounds^65^. Higher-order infection control circuits can be envisioned wherein AI-2-secreting host cells hijack the native quorum-sensing systems of bacteria without interfering with mammalian metabolism or communication. Our approach can address wild-type pathogenic microbes and thus contrasts with previous synthetic biology approaches wherein bacteria had to be artificially manipulated^68^.

Biosensor-based circuits in implanted human designer cells programmed to interface with infection-related molecules and to mitigate microbial colonization may pave the way for next-generation antimicrobials in the post-antibiotic era.

## Methods

### Components of the microbial-control device

Comprehensive design and construction details of the expression vectors are provided in Supplementary Data 1. The key components of the formyl-peptide-programmable microbial-control device consist of (i) a FPS module that detects formylated peptides, such as fMLF, by the corresponding human formylated peptide receptor FPR1 (pFS98 (P_SV40_-*FPR1*-pA; GenBank MF688634), (ii) the adapter protein Gα16, which redirects receptor signaling to the Ca^2+^ transduction pathway (pFS102 (P_SV40_-Gα16-pA; GenBank MF688633)), (iii) constitutively expressed 5′-methylthioadenosine nucleosidase MTAN (pFS84 (P_hCMV_-*mtaN*-pA; GenBank MF688635)) cleaving endogenous SAH and, (iv) the LuxS under the control of a Ca^2+^-responsive promoter (pFS186 (P_Ca2_-*luxS*-pA; GenBank MF688632)) to produce AI-2.

### Microbial strains

*Escherichia coli* strain XL10-Gold^®^ (XL10-Gold^®^ ultracompetent cells, Agilent Technologies, Basel, Switzerland; cat. no. 200314) was used for molecular cloning, for microbial composition experiments and plasmid propagation. *E. coli* strain RP437 (CGSC#: 12122; Coli Genetic Stock Center, Yale, USA) was used for AI-2 accumulation experiments and the ΔLuxS strain DH5α™ (Thermo Fisher, Basel, Switzerland; cat. no. 18265017) for cocultivation experiments. *E. coli* was grown at 37 °C on LB agar plates or in liquid LB medium (Beckton Dickinson, NJ, USA; cat. no. 244610) supplemented with appropriate antibiotics (Ampicillin, 100 µg/mL, cat. no. A9518; Kanamycin, 30 µg/mL, cat. no. K1377, both from Sigma-Aldrich, Munich, Germany). *Bacillus cereus* (ATCC: 10987) was cultivated in LB medium. *Streptococcus pneumoniae* (NCTC: 7466) was grown in THB medium containing 5% FCS, while *Staphylococcus aureus* (ATCC: BAA-1707), *Salmonella enterica subsp. enterica* (SGSC: 3580*), Enterococcus faecalis* (ATCC: 29212) and *Listeria innocua* (DSM 20649) were grown in MHB medium containing 5% FCS. *Lactobacillus reuteri* (DSMZ: 20016) was cultivated MRS medium. Plasmids for transfection were purified using a commercial kit (Genomed Jetstar 2.0 Midiprep, Genomed AG, Bad Oeynhausen, Germany). To detect AI-2, the *V. harveyi* strains BB170 (LuxN::Tn5, ATCC: BAA-1117), MM32 (luxN::Cm, luxS::Tn5Kan, ATCC: BAA-1121), TL26 (ΔluxN, ΔluxS, ΔcqsS)^56^ and BB120 (wild type, ATCC: BAA-1116) as well as JMH626^46^ were propagated at 30 °C in AB-Medium (0.3 M NaCl, 10 mM potassium phosphate, 1 mM l-arginine, 10 mM potassium phosphate, 0.1 mM arginine, 1% glycerol), in case of BB170, TL26 and MM32 supplemented with kanamycin (30 µg/mL). *Candida albicans* (ATCC: 10231-MINI-PACK) was cultivated in Yeast Extract-Peptone-Dextrose (YPD) medium at 30 °C and cell numbers were quantified optically at 600 nm with a Novaspec II photometer (Pharmacia, Freiburg, Germany). *P. aeruginosa* (PAO1, LasI^-^, PT466) was grown in LB medium at 37 °C.

### Cell culture and stable cell line generation

Human embryonic kidney cells (HEK-293T, ATCC: CRL-11268), human cervical carcinoma cells (HeLa, ATCC: CCL-2), human fibrosarcoma cells (HT-1080, ATCC: CCL-121), African green monkey kidney fibroblast-like cells (COS-7, ATCC: CRL-1651), human bone marrow stromal cells transgenic for the catalytic subunit of human telomerase (hMSC-TERT;^69^), human alveolar basal epithelial adenocarcinoma cells (A549, ATCC: CCL-185) and the human colorectal adenocarcinoma cell line (Caco-2, DSMZ: ACC 169) were cultivated in Dulbecco’s modified Eagle’s medium (DMEM; Thermo Fisher, Basel, Switzerland; cat. no. 52100-039) supplemented with 10% (v/v) fetal calf serum (FCS; Bioconcept, Allschwil, Switzerland; cat. no. 2-01F10-I; lot no. PE01026P) and 1% (v/v) penicillin/streptomycin solution. All cells were cultured at 37 °C in a humidified atmosphere containing 5% CO_2_.

All cells except for Caco-2 and A549 were transfected using an optimized polyethyleneimine (PEI)-based protocol. A transfection solution containing 2 µg of plasmid DNA mixtures, 10 µl PEI (PEI “max”, 1 mg/ml in water; Polysciences, Eppelheim, Germany; cat. no. 24765-2) was incubated for 20 min in 800 µl of DMEM at room temperature, then added dropwise to 2.5 × 10^5^ cells per well, seeded in a six-well plate the day before transfection. Before seeding, cell number and viability were evaluated using an electric field multi-channel cell counting device (Casy^®^ Cell Counter and Analyser Model TT; Roche Diagnostics GmbH, Basel, Switzerland). After cotransfection, the culture medium was replaced six hours after transfection for all cell lines (after seven hours for hMSC-TERT). Caco-2 and A549 cells were transfected for 48 h using Lipofectamine 3000/P3000 transfection according to manufacturer’s instructions (Thermo Fisher, Basel, Switzerland, cat. no. L3000001).

For the generation of double stable HEK-293-derived cell line HEK-293_sFS26c12_ transgenic for simultaneous constitutive P_EFIα_-driven MTAN and P_hCMV_-driven LuxS-eYFP expression, 250,000 cells were first transfected with 2000 ng of pFS168 (P_EFIα_-MTAN) and selected in culture medium containing 20 µg/ml Blasticidin (InvivoGen, San Diego, CA, USA, ant-bl-1) for 2 weeks. Following expansion of single clones by limiting dilution for another 2 weeks and validation of functional MTAN expression, the best performing clone (HEK-293_sFS25c21_) was co-transfected with 1660 ng of fluorescent S-Ribosylhomocysteinase (LuxS-eYFP; P_hCMV_-luxS-eYFP-pA, pFS169) and 340 ng of the zeocin resistance encoding plasmid pZeoSV2(+). After 17 days of selection in medium containing 200 µg/ml (w/v) zeocin (Invivogen, San Diego, CA, USA, cat. no. ant-zn-1) and 20 µg/ml Blasticidin, resistant monoclonal cells (HEK-293_sFS26cx_) were obtained by limiting dilution cloning and screened for AI-2 activity in their supernatants. The cell lines were regularly tested for the absence of *Mycoplasma*.

When generating the triple stable FPS_SEAP36_ transgenic for simultaneous constitutive P_EFIα_-driven FPR1, P_EFIα_-driven G_α16_ and P_Ca2_-driven SEAP expression, 2.4 × 10^5^ HEK-293 cells were sequentially engineered by co-transfecting pFS231 (ITR-P_Ca2_-SEAP-pA:P_RPBSA_-ZeoR-pA-ITR), pFS232 (ITR-P_hEF1α_-FPR1-pA:P_RPBSA_-BFP-P2A-PuroR-pA-ITR) and pFS233 (ITR-P_hEF1α_-G_α16_-pA:P_RPBSA_-tdTomato-P2A-BlaR-pA-ITR) with 10% (w/w) of the Sleeping Beauty transposase expression vector pCMV-T7-SB100 (P_hCMV_-SB100X-pA,), respectively. One day after transfection of pFS231, cells were selected for 10 days in culture medium containing 100 μg/ml zeocin and, after the second transfection (pFS232), for 14 days in culture medium additionally supplemented with puromycin (0.5 µg/ml). Triple stable clones resulting from pFS233 transfection of the double resistant polyclonal cell population were selected for 10 days with blasticidin (10 µg/ml) before stable cell clones were selected for tdTomato and BFP expression by FACS-mediated single-cell cloning. In a second step, the best-performing clone FPS_SEAP36_ was further engineered with an inducible AI-2 module encoded by pFS274 (ITR-P_hCMV_-MTAN-pA:P_RPBSA_-ZeoR-P2A-iRFP-pA-ITR) and pFS285 (ITR-P_Ca2_-LuxS-pA:P_RPBSA_-ZeoR-P2A-eGFP-pA-ITR) and cotransfected with SB transposase vector (pCMV-T7-SB100) before selecting penta-transgenic clones clones FPS_AI2-57_ via eGFP and iRFP expression using FACS-mediated single-cell cloning.

### FACS-mediated cell sorting

The HEK-293 cell subpopulation with top 10% expression of BFP (405 nm laser, 495 nm long-pass filter, 450/50 emission filter), and tdTomato (561 nm laser, 570 nm long-pass filter, 586/15 emission filter) and with top 1% GFP (488 nm laser, 505 nm long-pass filter, 529/28 emission filter) and iRFP expression (640 nm laser, 670 nm long-pass filter, 720/13 emission filter) were sorted using a Becton Dickinson LSRII Fortessa flow cytometer (Becton Dickinson, Allschwil, Switzerland). Dead cells and cell doublets were excluded and untreated HEK-293 cells or parental polyclonal populations served as negative controls.

### AI-2 quantification

Cells were cultivated in DMEM (Thermo Fisher, Basel, Switzerland, low glucose, without phenol red, cat. no. 11880-028) supplemented with 4 mM l-glutamine (Thermo Fisher, Basel, Switzerland, cat. no. 25030-024) and 1% FCS without antibiotics for 24 h (unless otherwise stated) and cell-free samples were prepared by centrifuging cell supernatants of transfected cells. To quantify AI-2 levels, a stationary overnight culture of *Vibrio* (BB120/BB170/JMH626) was grown in AB-Medium and diluted 1:5000 in sterile AB-Medium, while TL26 was diluted 1:1000. When using the LuxS^-^-deficient MM32 reporter strain, a stationary overnight culture grown in Luria Marine (LM)-medium^11^ was diluted 1:500 in AB-Medium and incubated for 1 h at 30 °C. Cell culture supernatants (10 µl) were analyzed by adding 90 µl of the respective *Vibrio* AI-2 reporter strain. *Vibrio* strains were grown for 3.5 h (BB170, BB120, JMH626), 6 h (MM32) or 8 h (TL26), respectively, in black-bottomed Fluotrac 200 96-well plates (Greiner Bio-One, Frickenhausen, Germany) at 30 °C and 200 rpm prior to measuring bioluminescence on a GENios Pro plate reader (TECAN AG, Maennedorf, Switzerland). Integration time was set at 1000 ms. Chemically synthesized DPD was purchased (Omm Scientific, Dallas, Texas, USA) and used as a positive control. Background luminescence of the negative control (AB-medium) was subtracted from samples. The DPD standard curve was calculated using GraphPad Prism 6 (GraphPad Software, CA, USA) and a four parameter logistic fitting.

### Sensing of formyl peptides from different microbes

*E. coli* (XL-10 Gold), *P. aeruginosa* (PAO1, PT466) and *V. harveyi* (BB170) in LB medium, *C. albicans* (ATCC: 10231) and *Saccharomyces cerevisiae* BY4741 (Euroscarf, Johann Wolfgang von Goethe-University Frankfurt am Main, Germany) in YPD medium, were grown overnight, washed, diluted (1:10) and resuspended in Roswell Park Memorial Institute 1640 culture medium (RPMI; Thermo Fisher, Basel, Switzerland; cat. no. 52400-025) and then grown for 3 h. Supernatants were collected by centrifugation, sterile-filtered and finally added to transiently transfected FPS_SEAP_ cell populations (40% v/v).

### Chemical compounds

Amphotericin B (25 mg/mL stock in DMSO Sigma-Aldrich, cat. no. A2411) was used for inhibition of fungal growth. l-norepinephrine (10 mM stock solution in DMSO; Sigma-Aldrich, cat. no. A7257), acetylcholine chloride (10 mM stock in DMSO, Sigma-Aldrich, cat. no. A2661), fMLF (10 mM stock in DMSO, Sigma-Aldrich, cat. no. 47729), fMIFL (10 mM stock in DMSO, Phoenix Pharmaceuticals, cat. no. 072-05), tBOC (2 mM stock solution in DMSO, Tocris, cat. no. 3730) and human serum (Sigma-Aldrich, cat. no. H4522) were directly added to FPS_SEAP_-engineered cells.

### Microbial biofilm formation assay

We used a modified MTT assay for biofilm quantification^9^ of *Bacillus cereus, Escherichia coli*, and *Candida albicans*. For testing the effect of exogenous AI-2, microbial strains inoculated (OD_600_ = 0.01) in microtiter wells or established *C. albicans* biofilms (24 h) were treated with in vitro synthetized DPD and biofilms were quantified 24 h later. For co-cultivation experiments, HEK-293 cells were seeded (250,000 cells/well) into the bottom of a Transwell (BD Biosciences, Sparks, MD, USA, cat. no. 353092), and transfected for 6 h before replacing the culture medium (10% FCS). Transwells inlets were placed into a six-well plate containing either mock-transfected control cells or cells harboring the microbial-control device for programmable AI-2 release and filled with 1 ml of medium. At 24 h after transfection, a stationary 24-hour culture of *C. albicans* grown in YPD medium was washed and resuspended in PBS prior to inoculating the upper Transwell with 400,000 cfu. Following another 24 h of co-cultivation, the culture medium in the upper culture chamber was replaced with fresh medium (DMEM, without phenol red) supplemented with MTT solution (Sigma-Aldrich, Munich, Germany; cat. no. M5655) at the final concentration of 0.5 mg/ml. After 1 h of incubation, the supernatants were carefully removed and the MTT dye in the upper chamber was solubilized in 1 ml acidic isopropanol/Triton mixture (0.1 N HCl, 10% (v/v) Triton X-100) for 15 min. Absorbance of the solubilized MTT dye was quantified optically at 550 nm with 650 nm background subtraction on an InfintePro multi-well reader (TECAN AG, Maennedorf, Switzerland).

### Analysis of microbial composition

To verify the effect of AI-secreting cells on the composition of mixed microbial communities, stationary overnight cultures of four microbial strains (*C. albicans, E. coli* XL10-Gold-pKDT17 (AmpR), *V. harveyi* MM32 (KanR) and *L. reuteri* 20016) were washed, normalized to OD_600_ = 0.01 and resuspended in AB medium. Engineered HEK-293 cells cultivated in modified DMEM (30% AB medium, 1% FCS) were infected with the microbial consortium (multiplicity of infection of each strain, MOI = 8). After 24 h, culture supernatants containing the microbial populations were serially diluted in LB (10^−1^/10^−6^) and plated on (semi-)selective agar plates for c.f.u. analysis. Colonies of *C. albicans* were determined on YPD plates supplemented with Chloramphenicol, *E. coli* on LB (Ampicillin) and *V. harveyi* on LB (Kanamycin) agar plates after 20 h at 30 °C. *L. reuteri* colonies were counted from MRS agar Vegitone plates (Sigma-Aldrich, cat. no. 41782) containing amphotericin B (2.5 µg/ml) for 24 h of incubation at 37 °C, 8% CO_2_.

### Western blot analysis

To check for full length eYFP-fusion protein expression in HEK-293 cells, western blot analysis was performed by resuspending 1 × 10^6^ HEK-293 cells in 100 µl RIPA lysis buffer (50 mM Tris–HCl (pH 8), 0.15 M NaCl, 1% (v/v) Triton X-100, 0.5% (w/v) sodium deoxycholate, 0.1% (w/v) SDS and a cocktail of protease inhibitors (Roche, Mannheim, Germany; cat. no. 11873580001) and constant agitation at 4 °C for 30 min. The lysate was cleared from cell debris by centrifugation (4000×*g* for 10 min at 4 °C) and the protein content of the supernatant was quantified (Bio-Rad protein assay; Bio-Rad Laboratories, Hercules, CA, USA; cat. no. 500-0006). Samples of 25 µg protein were mixed with 5× loading buffer (10% SDS, 1 M Tris pH 6.8, 50% glycerol, 0.2% bromphenol blue, 0.5 M DTT), resolved on a 12% SDS-PAGE and electroblotted (Trans-blot SD semi-dry transfer cell; Bio-Rad, Hercules, CA, USA) onto a polyvinylidene fluoride membrane (Immobilon-P, Millipore, Billerica, MA, USA; cat. no. IPVH00010). MTAN-eYFP and LuxS-eYFP fusions were visualized using a mouse anti-GFP antibody (sc-9996; lot. no. F2212, 1:1000) and a horseradish peroxidase-conjugated anti-mouse secondary antibody (GE Healthcare, Buckinghamshire, UK; cat. no. NA931V, lot no. 399402, 1:10,000) and ECL Plus Western blotting reagents (Amersham, GE Healthcare, Buckinghamshire, UK; cat. no. RPN2132). β-Actin served as a loading control (primary anti-actin antibody, Sigma-Aldrich, Munich, Germany; cat. no. A2066, lot no. 030M4844, 1:100; secondary horseradish peroxidase-coupled anti-rabbit antibody, AbD Serotec, Oxford, UK; cat. no. STAR54, lot no. 291010, 1:10,000). Chemiluminescence-based signal detection was performed with a Chemilux CCD camera (ImageQuant LAS 400 mini; GE Healthcare).

### Bacterial co-cultivation and bioluminescence imaging

*V. harveyi* and *E. coli* (DH5α) were grown overnight in LM and LB medium, respectively. Various host cell lines were seeded (63’000 cells/well) into the bottom of a 24-well permeable support companion plate (Corning, Lowell, MA, USA, cat. no. 353504), and transfected overnight. For *E. coli* infection assays, the culture medium was exchanged with 300 µl of fresh medium (DMEM; Thermo Fisher, Basel, Switzerland; cat. no. 52100-039) supplemented with 10% FCS in the lower compartment, or with 500 µl of fresh medium (DMEM, low Glucose, without phenol red, 1% FCS, 30 µg/ml kanamycin) for *Vibrio* assays. Transwells inlets (Corning, Lowell, MA, USA, cat. no. 353495) were placed on top of mock-transfected control cells or cells harboring the microbial-control device for programmable AI-2 release and inlets were filled with 0.20 ml containing 8 × 10^6^ *E. coli* CFUs in FCS-supplemented (4%) RPMI (Thermo Fisher, Basel, Switzerland; cat. no. 52400-025) and AI-2 levels in the lower compartment were quantified after 24 h. For *Vibrio* assays, inlets were filled with 250 µl AB medium spiked with 8 × 10^6^ CFU of *V. harveyi* (MM32). After 20 h of incubation at 30 °C, 5% CO_2_, bioluminescence of 100 µl samples taken from the upper compartment containing *V. harveyi* was quantified in black-bottomed Fluotrac 200 96-well plates (Greiner Bio-One, Frickenhausen, Germany) on a GENios Pro plate reader (TECAN AG, Maennedorf, Switzerland). Integration time was set at 1000 ms. Bioluminescence of the Transwell plate was additionally recorded with a Chemilux CCD camera (ImageQuant LAS 400 mini; GE Healthcare) with 1000 ms integration time.

### Resazurin viability assay

For monitoring viable cells with active metabolism, the resazurin assay was performed. In short, resazurin (Sigma-Aldrich, Munich, Germany; cat. no. R7017) was dissolved in culture medium (DMEM, 1% FCS, no phenol red) at 0.15 mg/ml and 20 µl was added directly to each well of a 96-well plate. The plate was incubated for 1 h (37 °C, 5% CO_2_) and fluorescence was measured with an InfintePro multi-well reader (TECAN AG, Maennedorf, Switzerland) at excitation and emission wavelengths of 560/9 nm and 590/20 nm, respectively. To calculate the percentage relative cell viability, the fluorescence of untreated cells was set to 100%.

### AI-2 inhibitor evaluation

The effectiveness and cytotoxicity of a MTAN transition state analog were profiled by seeding 25 000 HEK-293_AI-2(12)_ cells stably harboring pFS168 (P_EFIα_-MTAN-pA) and pFS169 (P_hCMV_-luxS-eYFP-pA) per well of a black 96-well µClear-bottom plates (Greiner Bio-One, Frickenhausen, Germany, cat. no. 7.655 090). After 16 h, cells were exposed to compounds for 24 h before scoring AI-2. To this end, an overnight culture of *V. harveyi* (MM32; LuxS^-^) grown in LM-medium was diluted 1:500 into AB-Medium (see AI-2 quantification for details), and 60 µl per well was dispensed. Plates were incubated for 6 h on a Heidolph Titramax shaker (VWR, Dietikon, Switzerland), at 30 °C at 200 rpm prior to *Vibrio* bioluminescence measurement on a M1000 Pro Reader (TECAN AG, Maennedorf, Switzerland) with 1 s integration time.

### Fluorescence and confocal imaging

Time-lapse fluorescence microscopy was performed with an inverted fluorescence microscope (DMI 6000B; Leica Microsystems) equipped with an incubation chamber, a DFC350FX R2 digital camera (Leica), a 10× objective (objective HC PL FL 10×/0.30 PH1 –/D 11.0; Leica), a 495/535-nm (Citrine) excitation/emission filter set, a Fluorescence LED light source (SFL7000, Leica) and LAS AF imaging software (FW4000-TZ; Leica). Images were taken every 10 min. Mean Citrine fluorescence was quantified using the software Fiji containing the Time Series Analyzer Plugin V3 (2014). Identical settings including exposure times of 200 ms for Citrine were used for all fluorescence micrographs. Single-time-point fluorescence microscopy was done on a fluorescence microscope (Nikon Eclipse Ti) using a fiber illuminator (Nikon Intensilight C-HGFI), optimized optical filtersets (Semrock) and a digital camera system (Hammamatsu, ORCA Flash 4). The filter set contains a combination of excitation bandpass filter, emission bandpass filter and a dichroic filter. We measured Citrine with 200 ms exposure time and a Citrine filter set: HC 470/40, HC 520/35, BS 495.

For high-resolution imaging, HEK-293 cells were seeded on LabTekII glass chamber slides (Cat.Nr.154526, Nunc, DK) and transiently transfected; nuclei were stained with 2.5 µg/ml of Hoechst 33342 (Fisher Scientific, MA, USA) 24 h later. Confocal micrographs of AI-2-producing cells expressing eYFP-fusion enzymes were recorded on a Leica TCS-SP5 confocal microscope (Leica Microsystems, Glattbrugg, Switzerland) equipped with a HCX PLAN APO 63.0 × 1.4 cs2 oil immersion objective, a 488-nm/509-nm laser for eYFP excitation and a 500-nm/600-nm emission filter set. Hoechst staining was imaged with a 405-nm laser for excitation combined with a 450-nm/500-nm emission filter set. Image analysis was done using the Leica Application Suite software (version 3.2.0).

### FACS analysis

Transfected and mock-transfected HEK-293 cells were analyzed using a Becton Dickinson LSRII Fortessa flow cytometer (Becton Dickinson, Allschwil, Switzerland) equipped for GFP (488 nm laser, 505 nm longpass filter, 586/15 emission filter) and DRAQ7™ (633 nm laser, 750 nm longpass filter, 780/60 emission filter) detection. Cells were gated to exclude cell doublets, dead cells and cell debris. At least 50,000 cells were recorded per data set and analyzed using FlowJo-877 software. (Tree Star Inc., Ashland, OR, USA, version no. 7.6.3.). Viability was quantified using the far-red dead cell dye DRAQ7™ in a final concentration of 1.5 µM (BioStatus, Leicestershire, UK).

### SEAP assay

Human placental SEAP activity in the cell supernatant was quantified as follows: 100 μL of cell culture supernatant was heat-inactivated for 30 min at 65 °C. Then, 40 μL of the supernatant and 40 µl water were transferred to a well of a 96-well plate containing 100 μL 2× SEAP assay buffer (20 mM homoarginine, 1 mM MgCl2, 21% diethanolamine pH 9.8). After addition of 20 μL 120 mM *para*-nitrophenyl phosphate (pNPP disodium salt, hexahydrate, Acros Organics BVBA, Geel, Belgium; cat. no. 12886-0100) diluted in 1 × SEAP assay buffer, the time-dependent increase in light absorbance was profiled at 405 nm for 30 min at 37 °C on an InfintePro multi-well reader (TECAN AG, Maennedorf, Switzerland).

### Data availability

All data and materials are available upon request. Sequences of key expression vectors have been deposited in GenBank: pFS83, MF688636; pFS84, MF688635; pFS98, MF688634; pFS102, MF688633 and pFS186, MF688632.

## Electronic supplementary material

Supplementary Information

Description of Additional Supplementary Files

Supplementary Data 1
