# Supplementary material for: Designer cells programming quorum-sensing interference with microbes
Source: Nat Commun. 2018 May 8;9:1822. doi: 10.1038/s41467-018-04223-7 (PMC5940823; doi:10.1038/s41467-018-04223-7)
Supplement: Supplementary file 1 — Supplementary Information [file 41467_2018_4223_MOESM1_ESM.pdf]

Supplementary Information

Designer cells programming quorum-sensing interference with microbes

Ferdinand Sedlmayer[1], Dennis Hell[1], Marius Müller[1], David Ausländer[1] and Martin Fussenegger[1,2*]

[1]Department of Biosystems Science and Engineering, ETH Zürich, Mattenstrasse 26, CH-4058 Basel, Switzerland, [2]Faculty of Science, University of Basel, Mattenstrasse 26, CH-4058 Basel, Switzerland.

**To whom correspondence should be addressed: Tel.: +41 61 387 31 60, Fax: +41 61 387 39 88, E-mail: fussenegger@bsse.ethz.ch*

Supplementary Figures

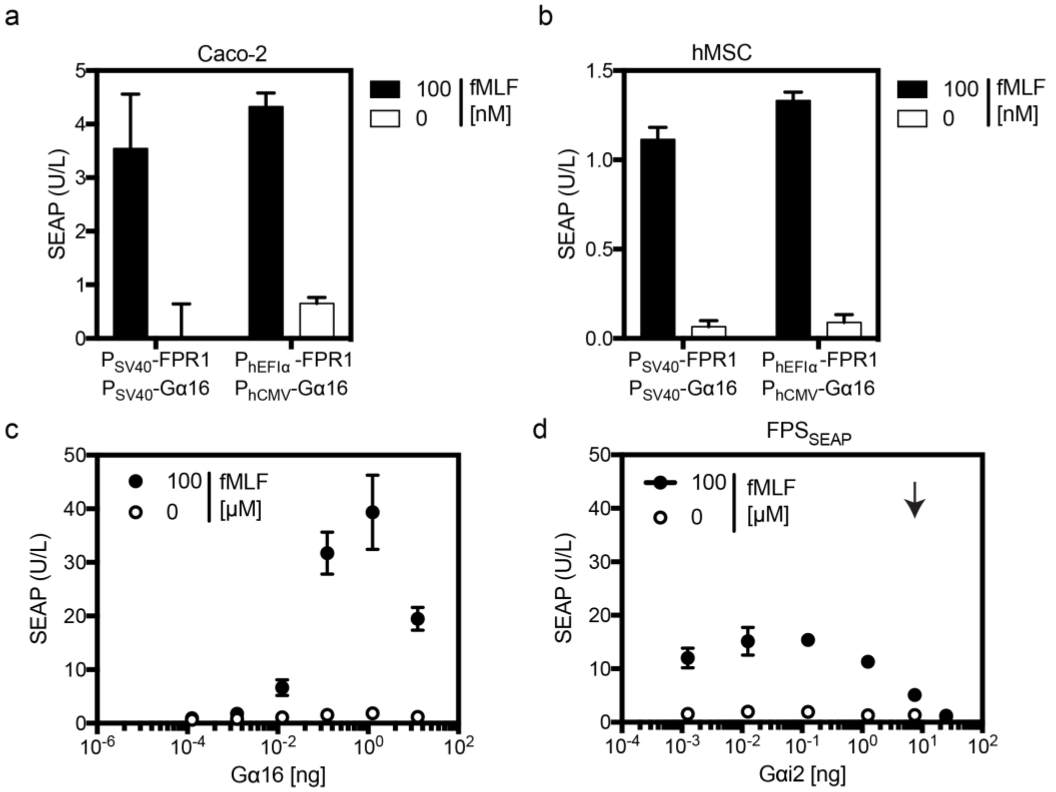

Supplementary Figure 1 | FPS circuit fine-tuning experiments. (a, b) Promoter strength optimization. (a) Caco-2 or (b) hMSC-TERT were cotransfected with weak P_{SV40}-driven vectors (pFS98/pFS102) or stronger $P_{PhEFI\alpha}$ or P_{hCMV}-driven constructs (pFS115/pcDNA3.1-$G_{\alpha16}$) in combination with the reporter pYL1 and challenged with formylated peptides for 24 h before SEAP levels were quantified. (c) Fine-tuning of Gα16 for FPR1 rerouting. HEK-293 cells cotransfected with pFS98/pYL01 plus increasing amounts of pcDNA3.1-Gα16 were exposed to fMLF for 24 h. (d) Competition between Gα16 and Gαi for FPR1 binding. FPS_{SEAP} cells (pFS98/pcDNA3.1-Gα16/pYL1) endogenously expressing Gαi proteins were co-transfected and titrated with pcDNA3.1-Gαi2 for ectopic Gαi2 overexpression. The arrow indicates an equimolar vector amount of P_{hCMV}-Gαi2 relative to P_{hCMV}-Gα16. The amount of P_{hCMV}-Gα16 was kept constant. (IC_{50}(Gαi2) = 4.8 ng). SEAP expression induced by fMLF was quantified after 24 h. Data show means ± SD (n = 3).

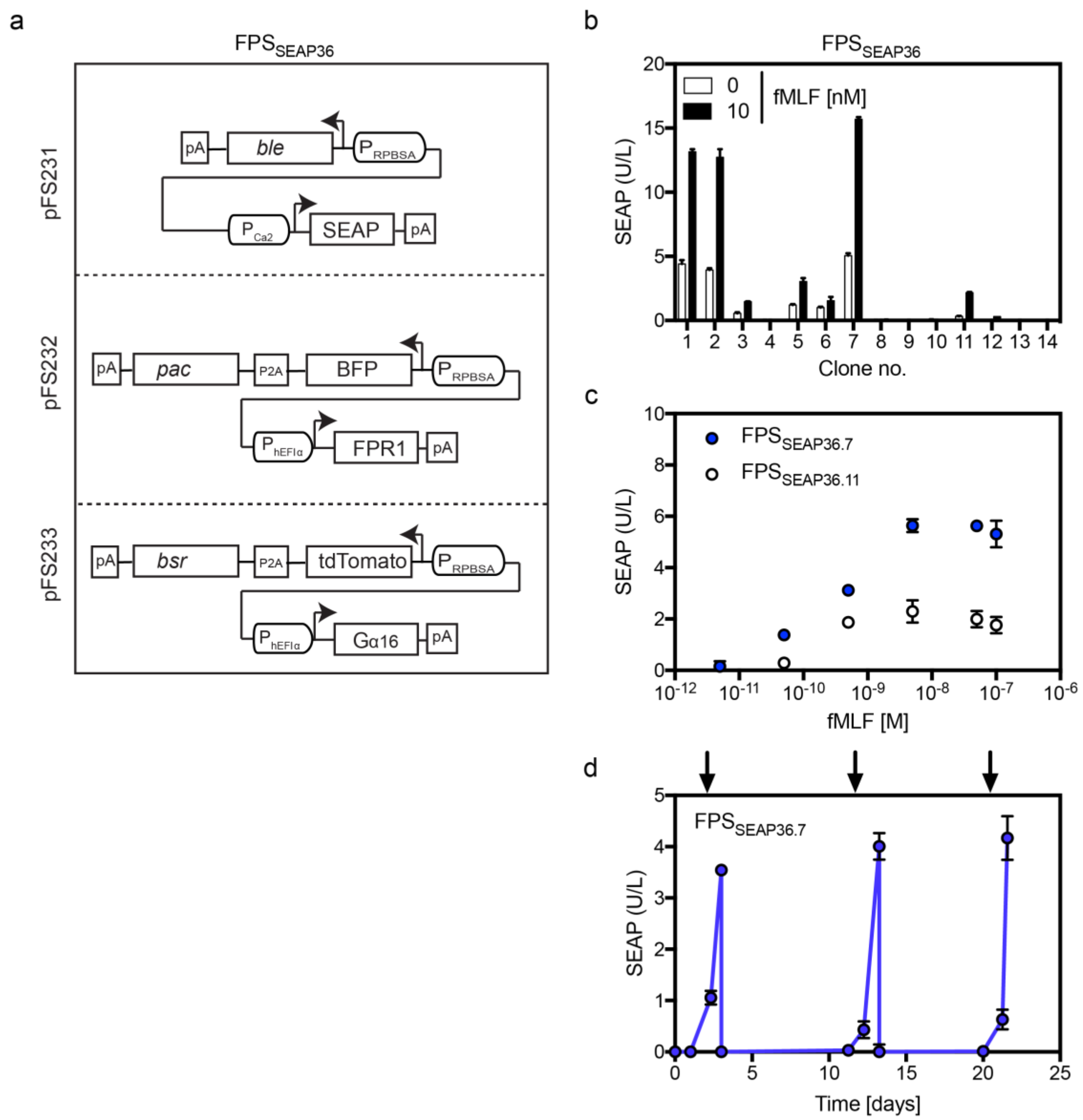

Supplementary Figure 2 | Production and characterization of stable cell lines for formyl peptide detection. (a) Illustration of genetic formyl peptide sensing components for stable chromosomal integration in FPS_{SEAP36} cells (see Supplementary Table 1 for details) (b) Performance of monoclonal HEK-293 cell populations harboring transposon-based FPS components (pFS231/pFS232/pFS233). Triple stable FPS_{SEAP36} cell populations with individual growth profiles were expanded and then cultivated for 24 h in the presence or absence of 10 nM fMLF to screen for only highly sensitive clones before SEAP was profiled in the culture supernatant. (c) fMLF sensitivity of monoclonal FPS_{SEAP36} cell populations (2.5×10^4 cells). (d) Long-term performance of FPS. $FPS_{SEAP36.7}$ cells were cultivated for 23 days, repeatedly induced for 1 h using fMLF (10 nM; arrows) and SEAP expression was profiled after 24 h. Data show means ± SD.

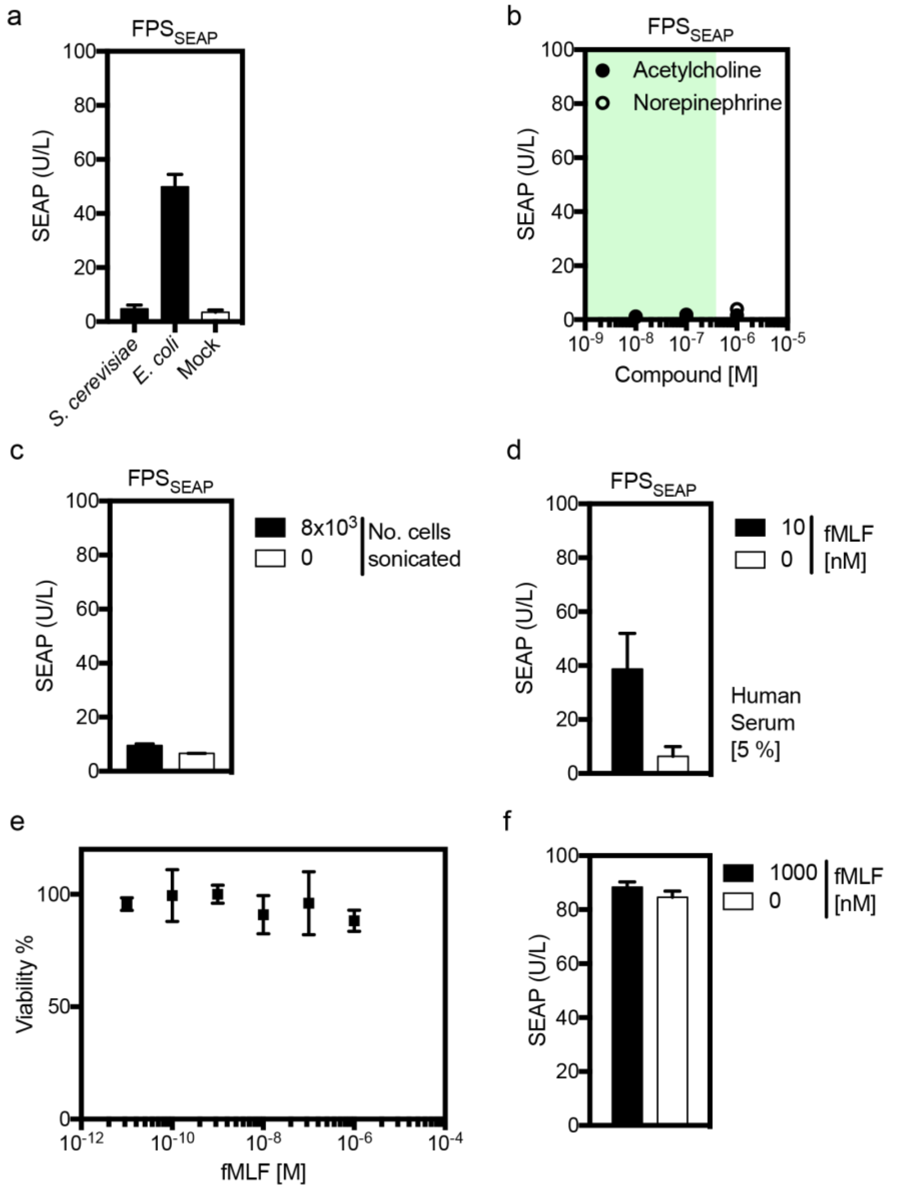

Supplementary Figure 3 | FPS specificity and viability impact of formylated peptides. (a) Formyl peptide-inducible responses to supernatants of *Saccharomyces cerevisiae* versus *Escherichia coli* of transiently FPS$_{SEAP}$-engineered cell populations after 24 h. (b) Specificity of the synthetic Ca^{2+}-based FPS signaling cascade. HEK-293 cells express endogenous Gq-coupled GPCRs such as the M1 muscarinic acetylcholine receptor (M1AchR) and the alpha-1B adrenergic receptor (ADRA1B) which could cross-talk with the Ca^{2+}-based signaling pathway when activated by norepinephrine, or acetylcholine, respectively. FPS$_{SEAP}$-cotransfected cells were supplemented with norepinephrine and acetylcholine (physiological concentrations represented by green translucent background) and cultivated for 24 h. (c) Sensitivity of the FPR1 receptor to mitochondrial formylated peptides. Transiently engineered FPS$_{SEAP}$ cells were reseeded onto 96-well plates and exposed to supernatants from sonicated or mock-treated HEK-293 cells for 24 h before determining a qualitative influence on

the FPS. (d) FPS_{SEAP}-engineered cells were supplemented with human serum (5%) and exposed to fMLF for 24 h before SEAP activity was scored. (e, f) Influence of formyl peptides on HEK-293 cell viability and SEAP production. Viability after 24 h fMLF exposure was measured via (e) resazurin fluorescence and the viability was calculated in relation to untreated cells and (f) through quantifying the productivity of pSEAP2-control-transfected cells. Data show the means ± SD of three independent experiments measured in triplicates.

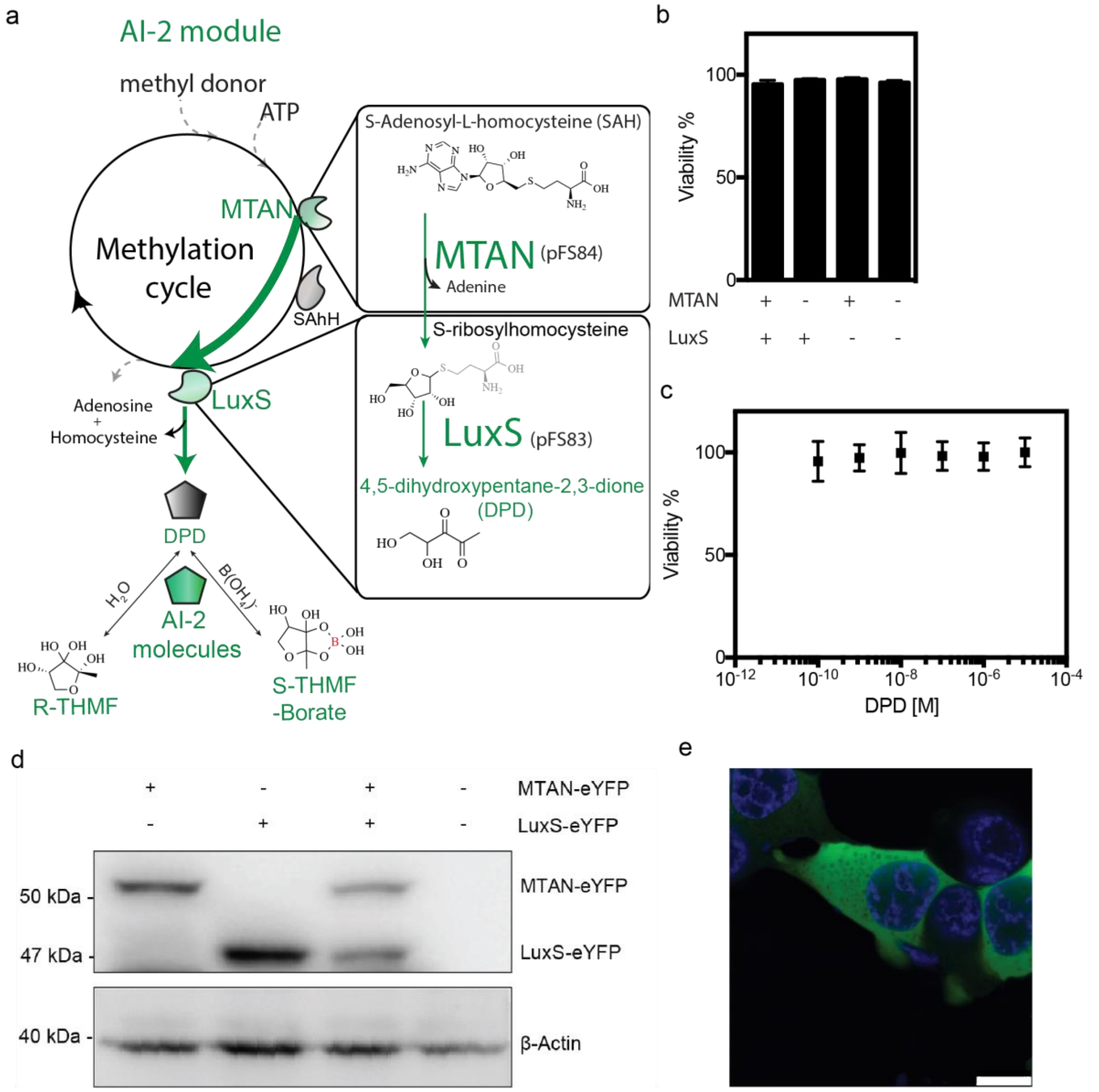

Supplementary Figure 4 | Viability impact of the AI-2 module components. (a) Illustration of the artificial methylation cycle by-pass, in which *E. coli*-derived MTAN intercepts the methionine cycle by competing with the endogenous S-adenosylhomocysteine hydrolase (SAhH) to convert the ubiquitous substrate S-adenosyl-homocysteine into S-ribosyl-homocysteine, which is further processed into 4,5-dihydroxypentane-2,3-dione (DPD) by LuxS. DPD afterwards interconverts into a family of signaling molecules known as AI-2. (b) Effect of ectopic expression of the genetic components for AI-2 production on cell viability. 24 h after co-transfection with MTAN (pFS84) and/or LuxS (pFS83) encoding expression vectors or EGFP (pEGFP-N1) HEK-293 cells were analyzed by FACS using the DRAQ7™ viability dye. (c) Impact of exogenous AI-2 precursor (4,5-dihydroxy-2,3-pentanedione, DPD)

on HEK-293 cells. Viability was measured via resazurin fluorescence after 24 h of incubation and normalized to untreated cells. Data show the means ± SD (n=3). (d) GFP-specific Western blot analysis of pFS169/pFS170-cotransfected HEK-293 cells expressing MTAN-eYFP and LuxS-eYFP alone or together (+/−). Actin was used as the loading control. (e) Cell morphology of fluorescent AI-2 secreting cells. Micrograph taken 24 h after co-transfecting HEK-293 cells with P_{hCMV}-LuxS-eYFP (pFS169) and P_{hCMV}-MTAN-eYFP (pFS170). Nuclear staining with Hoechst 33342. Scale bar=10 µm.

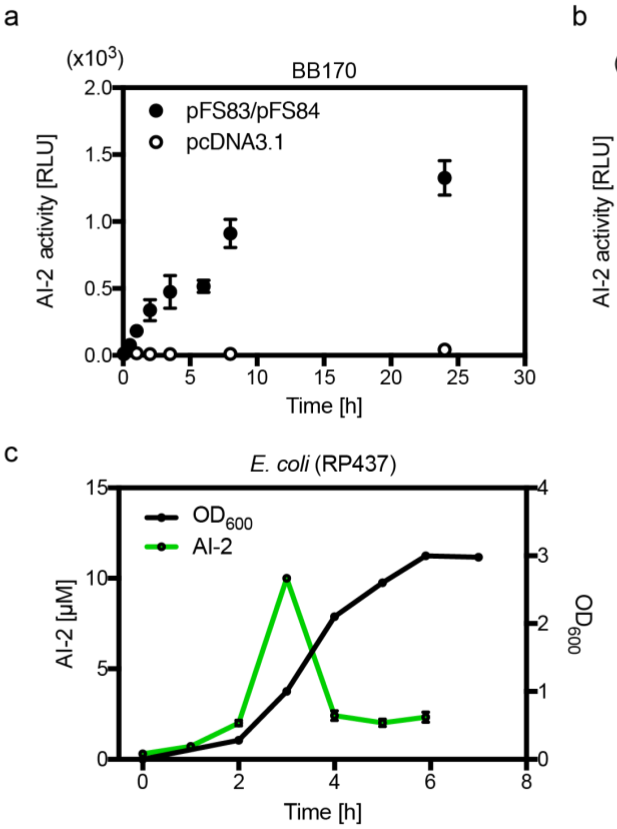

Supplementary Figure 5 | Validation and quantification of AI-2 release from mammalian and bacterial producers. (a) AI-2 release kinetics of HEK-293 cells engineered for continuous AI-2 secretion. After culture medium exchange, AI-2 accumulation in the supernatant of AI-2 (pFS83/pFS84) or Mock (pcDNA3.1)-engineered cells was repeatedly profiled through *V. harveyi* (BB170) bioluminescence during 24 h. (b) AI-2 quantification in cell supernatants after 24 h (blue circle) by *V. harveyi* (BB170) bioassay. AI-2 concentrations were calculated based on synthetic AI-2 standards (4,5-dihydroxy-2,3-pentanedione, DPD) spiked into conditioned culture medium (white squares). (c) AI-2 release from *E. coli* (RP437) during logarithmic growth. AI-2 from culture supernatants (LB) was sampled in regular intervals and absolute concentrations were calculated based on DPD reference bioluminescence of *V. harveyi*. AI-2 levels were plotted against *E. coli* growth (optical density readings at 600 nm). Data show the means ± SD of three independent experiments measured in triplicate.

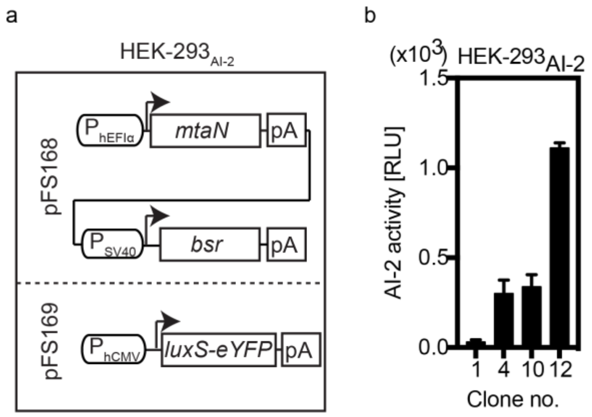

Supplementary Figure 6 | Design and validation of stable AI-2 producing cell populations. (a) Schematic illustration of genetic components from HEK-293$_{AI-2}$ cells for continuous AI-2 secretion (see plasmid table S1 for details). (b) AI-2 activity in supernatants of selected monoclonal cell populations scored by *V. harveyi* BB170. Data show the means ± SD of three independent experiments measured in triplicate.

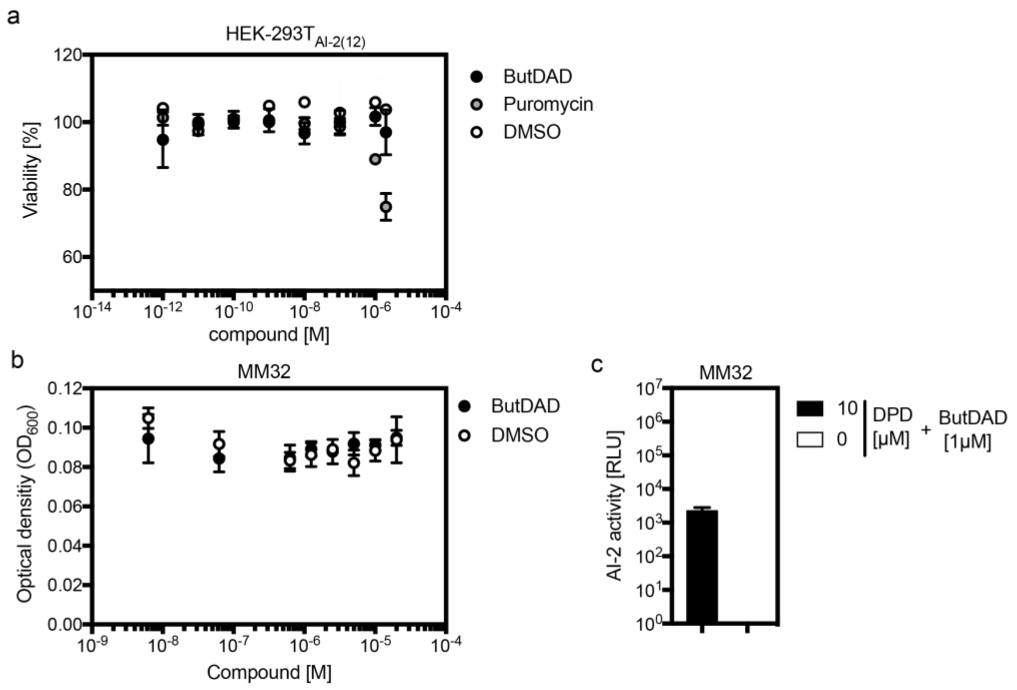

Supplementary Figure 7 | ButDAD influence on viability and AI-2 detection. (a) Resazurin viability assay of MTAN/LuxS-transgenic HEK-$293_{AI-2(12)}$ following MTAN inhibitor treatment (ButDAD) for 24 h. (b) Bacterial growth (*V. harveyi* MM32) in the presence of different ButDAD concentrations quantified by optical density readings at 600 nm. (c) Influence of ButDAD on *V. harveyi* AI-2 detection. Cell culture medium containing ButDAD was spiked with DPD (0 or 10 µM). Bioluminescence from *V. harveyi* (MM32) was subsequently quantified. Data represent means ± SD, n=3.

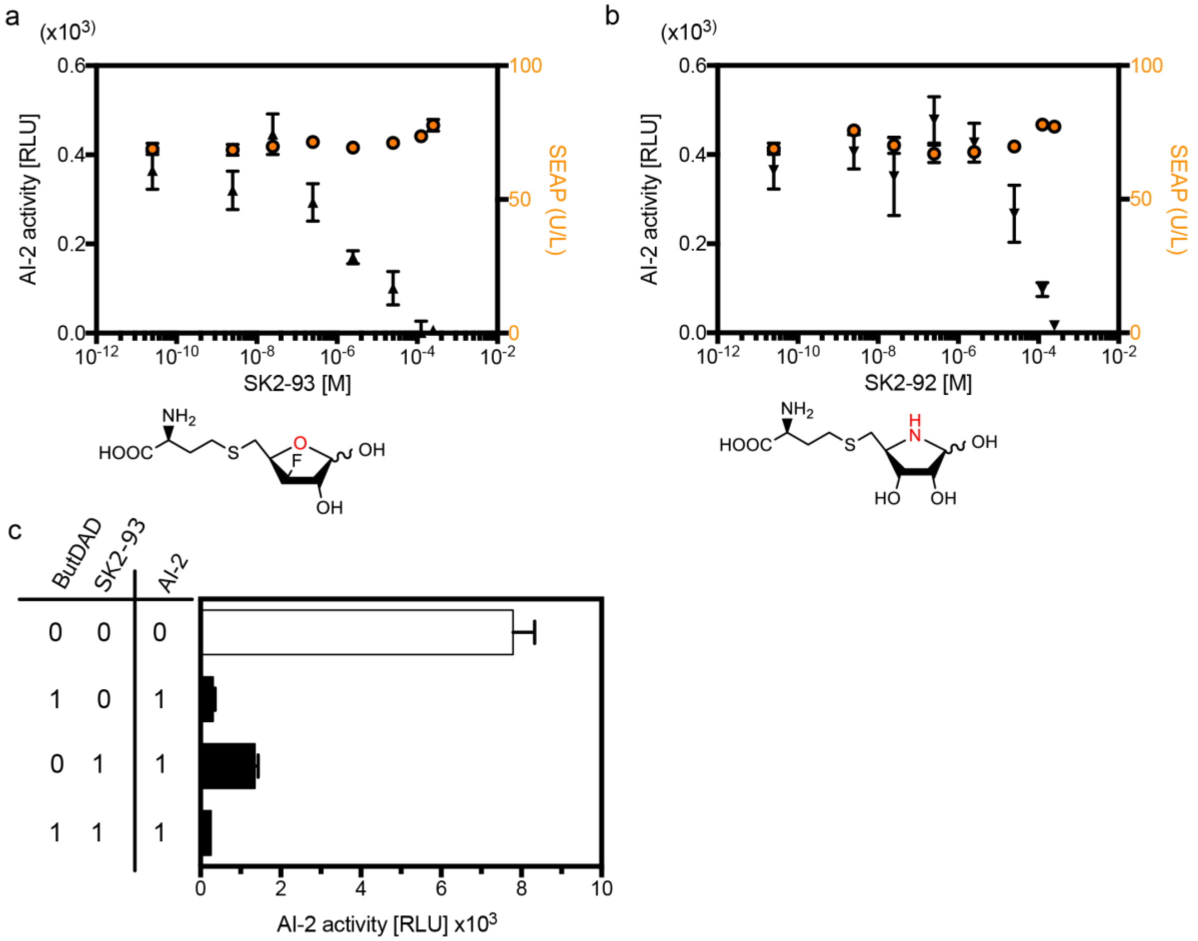

Supplementary Figure 8 | Cell-based LuxS inhibitor evaluation. Monitoring of AI-2 biosynthesis inhibition by the rationally designed LuxS inhibitors (a) SK2-93 or (b) SK2-92 or (c) SK2-93 [100 µM] in combination with the MTAN inhibitor ButDAD [100 nM]. AI-2/SEAP-engineered (pFS83/pFS84/pSEAP2-Control) HEK-293 cells were exposed for 24 h to different compounds before SEAP and AI-2 activity were assessed in culture supernatants. AI-2 was quantified by BB170 bioluminescence. Data show the means ± SD of triplicate experiments (n=3).

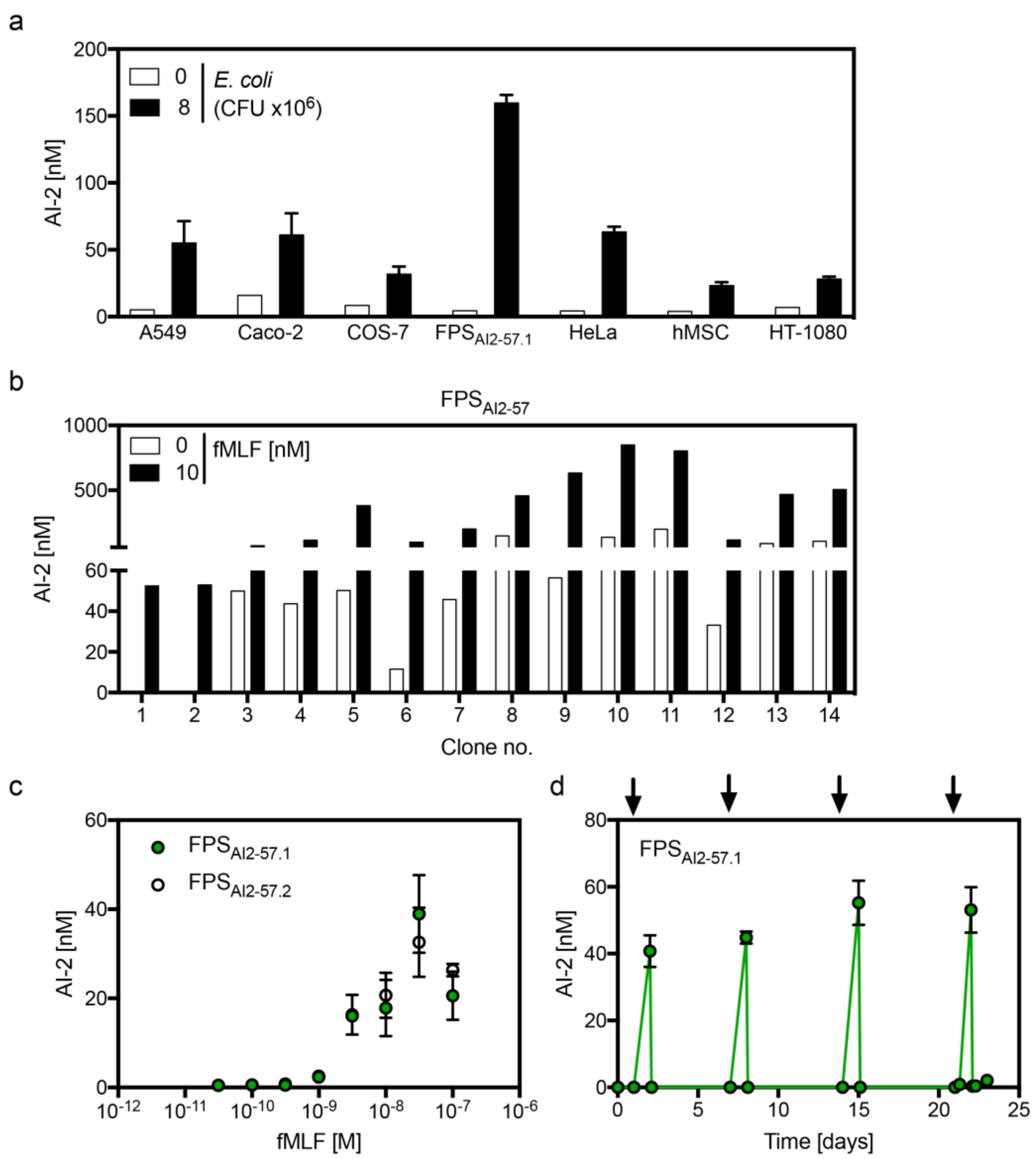

Supplementary Figure 9 | Programmable AI-2 release from different mammalian cell lines. (a) Infection response of cell lines transiently engineered with the microbial-control circuit (pFS115/pDH1/pFS170/pFS186) or of the HEK-293-derived stable monoclonal penta-transgenic (pFS231/pFS232/pFS233/pFS274/pFS285) $FPS_{AI2-57.1}$. Transfected cells were grown in cell-culture dishes and exposed to *E. coli* grown in Transwells® for 24 h before AI-2 levels were quantified. (b) Performance of stable monoclonal FPS_{AI2} cell populations. Penta-transgenic FPS_{AI2-57} cell populations with individual growth profiles were expanded and then cultivated for 24 h in the presence or absence of 10 nM fMLF before AI-2 levels were profiled in the culture supernatant. (c) Individual FPS_{AI-2}-derived monoclonal penta-transgenic cell populations with minimal leakiness were exposed to different concentrations of formylated peptides for 24 h before AI-2 levels were measured in cell supernatants. (d) Long-term stability and responsiveness of the microbial-control device. $FPS_{AI2-57.1}$ cells were cultivated for 23

days, repeatedly induced for 1 h by fMLF exposure (10 nM; arrows) and AI-2 levels were profiled after 24 h. Data show the means ± SD (n=3).

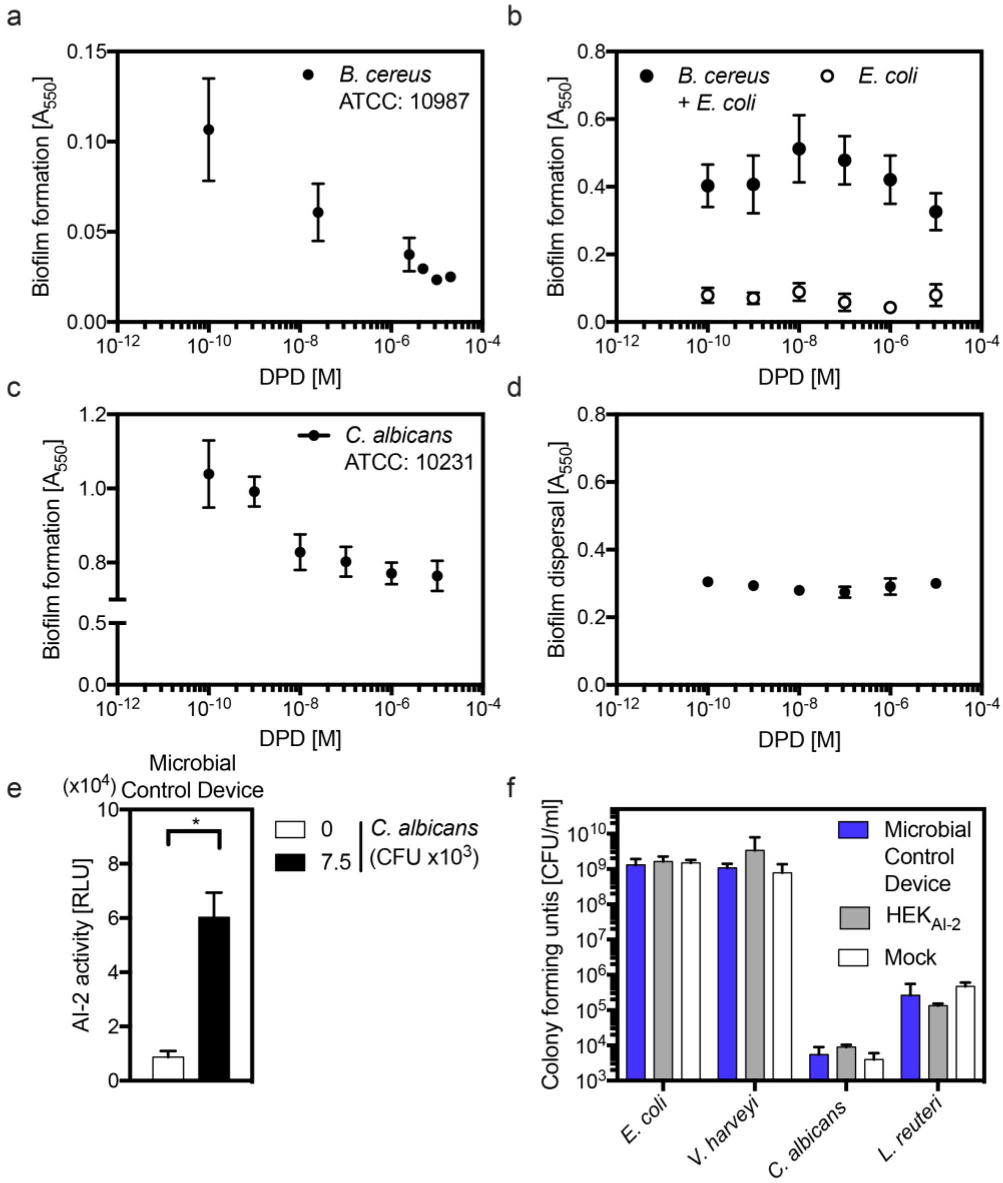

Supplementary Figure 10 | AI-2 and microbial-control circuit influence on microbial strains and communities. Autoinducer-2 (DPD) influence on biofilm formation of (a) *Bacillus cereus*, (b) dual species communities including *Escherichia coli* and *B. cereus* (c) or *Candida albicans*. Metabolic activity of microbial biofilms following DPD exposure was measured through MTT after 24h. (d) AI-2 influence on established (24 h preformed) *C. albicans* biofilms measured after 24 h. (e) *C. albicans*-programmable AI-2 release. Microbial-control-components-cotransfected HEK-293 cells were grown in the bottom chamber of a Transwell and then challenged with *C. albicans* in the upper compartment for 24 h before AI-2 levels in cell supernatants were measured using the *V. harveyi* (MM32) bioassay. (f) Microbial-control, AI-2 or Mock ($FPS_{Citrine}$)–engineered HEK-293 cells were cocultivated with

an AI-2 responsive quadruple-species microbial community for 24 h (MOI = 8). Microbial compositions were assessed by counting CFUs from selective agar plates. Data show the means ± SD of three independent experiments measured in triplicates.

Supplementary Tables

Supplementary Table 1. Plasmids and oligonucleotides used and designed in this study

See 'Supplementary Data 1'

Supplementary Table 2. Microbial densities for sensing formylated peptides by the FPS

Strain	OD_{600}	Conversion factor CFU/ml per 1 U OD_{600}	CFU before sample filtration (100 uL)
B. cereus	0.1	0.8×10^9	8.00E+06
P. aeruginosa	0.1	0.8×10^9	8.00E+06
E. coli	0.1	0.8×10^9	8.00E+06
S. pneumoniae	0.1	0.8×10^9	8.00E+06
S. aureus	0.1	0.8×10^9	8.00E+06
S. enterica	0.1	0.8×10^9	8.00E+06
E. faecalis	0.1	0.8×10^9	8.00E+06
L. innocua	0.1	0.8×10^9	8.00E+06
V. harveyi	0.1	0.8×10^9	8.00E+06
S. cerevisiae	0.87	3×10^7	2.61E+06
C. albicans	0.43	3×10^7	1.29E+06
