# Supplementary material for: Designer cells programming quorum-sensing interference with microbes
Source: Nat Commun. 2018 May 8;9:1822. doi: 10.1038/s41467-018-04223-7 (PMC5940823; doi:10.1038/s41467-018-04223-7)
Supplement: Supplementary file 2 — Description of Additional Supplementary Files [file 41467_2018_4223_MOESM2_ESM.pdf]

Description of Additional Supplementary Files

File Name: Supplementary Data 1
Description: The plasmid and oligonucleotide list, describing the construction of the genetic components used in this study
